# Generation of a mouse SWATH-MS spectral library to quantify 10148 proteins involved in cell reprogramming

**DOI:** 10.1038/s41597-021-00896-w

**Published:** 2021-04-26

**Authors:** Uxue Ulanga, Matthew Russell, Stefano Patassini, Julie Brazzatti, Ciaren Graham, Anthony D. Whetton, Robert L. J. Graham

**Affiliations:** 1 Clinical Proteomics Research Group, Division of Molecular and Clinical Cancer Sciences, Faculty of Biology, Medicine and Health, University of Manchester, Oxford Road Manchester, Manchester, UK; 2 Stoller Biomarker Discovery Centre, Division of Molecular and Clinical Cancer Sciences, NIHR Biomedical Research Centre, Faculty of Biology, Medicine and Health, University of Manchester, Oxford Road Manchester, Manchester, UK; 3 Stem Cell & Leukaemia Proteomics Laboratory, Manchester Cancer Research Centre, Division of Molecular and Clinical Cancer Sciences, Faculty of Biology, Medicine & Health, University of Manchester, Manchester, UK; 4 Centre for Biosciences, School of Healthcare Science, Manchester Metropolitan University, Manchester, UK; 5 School of Biological Sciences, Queen’s University Belfast, Chlorine Gardens, Belfast, UK

**Keywords:** Induced pluripotent stem cells, Reprogramming, Proteomics, Proteome informatics, Mass spectrometry

## Abstract

Murine models are amongst the most widely used systems to study biology and pathology. Targeted quantitative proteomic analysis is a relatively new tool to interrogate such systems. Recently the need for relative quantification on hundreds to thousands of samples has driven the development of Data Independent Acquisition methods. One such technique is SWATH-MS, which in the main requires prior acquisition of mass spectra to generate an assay reference library. In stem cell research, it has been shown pluripotency can be induced starting with a fibroblast population. In so doing major changes in expressed proteins is inevitable. Here we have created a reference library to underpin such studies. This is inclusive of an extensively documented script to enable replication of library generation from the raw data. The documented script facilitates reuse of data and adaptation of the library to novel applications. The resulting library provides deep coverage of the mouse proteome. The library covers 29519 proteins (53% of the proteome) of which 7435 (13%) are supported by a proteotypic peptide.

## Abstract (summary)

**Table:** 

Measurement(s)	database type spectral library • protein expression data
Technology Type(s)	liquid chromatography-electrospray ionisation time-of-flight mass spectrometry • SWATH MS protein profiling assay
Factor Type(s)	treatment • cell development stage
Sample Characteristic - Organism	Mus musculus

Machine-accessible metadata file describing the reported data: 10.6084/m9.figshare.14135576

## Background & Summary

Sequential window acquisition of all theoretical mass spectra (SWATH-MS) is a data-independent acquisition mode for quantitative proteomics^1–4^. SWATH-MS enables acquisition of liquid chromatography based mass spectrometry (LC-MS) data for multiple biological samples. The data is then queried against a spectral library containing assays comprised of spectra-retention time points from which quantitative elution profiles may be reconstructed. This schema enables reproducible whole proteome quantification across multiple samples affording insight into the protein driven cellular processes that direct the behaviour of cells and tissues^5^. This process generates a map of the whole proteome which can subsequently be reanalysed to test new hypothesis^6^.

The spectral library containing the spectral and retention time coordinates required to extract quantitative data from SWATH maps is therefore a critical component of such an experimental schema. Alternative DIA data processing strategies that directly interrogate SWATH maps without reference to spectral libraries have been developed but are generally less sensitive^7,8^. It is possible to generate project specific spectral libraries at the cost of considerable investment of sample preparation and instrument time, which never-the-less have the advantage of being obtained on precisely the same equipment used to generate SWATH maps. Large community shared spectral libraries on the other hand provide a shared set of reference spectra ready to apply to a sample set^9^.

Murine models are among the most widely used systems to understand biology and disease. At time of drafting there were no readily re-usable spectral libraries for this species, however there are now two; one build by Zhong *et al*.^10^ and another by Krasney *et al*.^11^A further mouse spectral library derived from tissue samples from heavy vascularised organs (e.g. liver, kidney, lung, heart or plasma) has previously been published^12^ but is not hosted on SWATHatlas. None of these libraries comprehensively cover the protein markers for iPSC reprogramming.

Other publicly accessible libraries available from the SWATHatlas website include several human libraries;^13^ Rat in the form of Sertoli cells; Zebrafish^9^; and several pathogens including *S. cerevisiae*^14^, *M. tuberculosis*^15^ and *S. pyogenes* proteome library^16^

We have built a spectral library^17^ for mouse cells in order to study the process by which primary mouse embryonic fibroblasts (MEFs) are reprogramed to induced pluripotent stem cells (iPSCs) by the addition of four genes^18^. By the nature of the requirement the library has to have extensive coverage of proteins. iPSCs are a powerful tool for modelling and investigation of diseases, drug discovery and toxicology research. The cells have the potential to transform healthcare by providing an unlimited source of cells for personalised therapy. Since their discovery questions including what cell types can become iPSCs, what iPSCs can be differentiated into, virus-free iPSC generation methods, differences between ESCs and iPSCs, how to improve the efficiency of iPSC generation or the molecular mechanisms involved in cellular reprogramming remain unanswered. However, the efficiency of cell reprogramming is low and somatic cells undergoing reprogramming are heterogeneous cells^19^. In addition, there is limited availability of markers to select intermediate populations prone to reach pluripotency^20^. Few authors have attempted to study the entire reprogramming course at genetic^21^ and protein level^22^.

A spectral library derived from the cells over a reprogramming time course was required to enable quantification of all relevant proteins involved in the reprograming process and to develop new tools for understanding the quality and mechanism of reprogramming enhancing previous libraries, for example the published mouse library derived from vascularised tissues (likely to be deficient in known markers of reprogramming including Col5a2, Fibrillin2, Thy1, Oct4, Nanog, Esrrb and Fbx15^20^).

However, the existence of this data set for mouse presented the opportunity to combine newly acquired cell derived data with the tissue data through a single processing pipeline to create a combined library, which might find wide use in the community. We therefore present four individual libraries covering the different temporal points in the production of iPSCs; a separate re-build of the mouse tissue library using our pipeline; and a “combined library” integrating all five data sets. We include a comprehensive documented processing script that should enable repeatable production of the library from the raw data with optional variations to support specific applications. Furthermore, the script is an example of the integration of data from multiple sources and we hope it provides a basis for future data sets to be integrated with those presented here to further increase the depth of coverage of this shared community resource.

## Methods

### NIH3T3

The NIH3T3 cells were cultured in DMEM media supplemented with 10% FBS (Gibco, USA), 100 U/ml of penicillin and streptomycin, 2 mM L-glutamine (Sigma, USA) and 0.1 mM non-essential amino acids (NEAA) (Gibco, USA) under 5% CO_2_ at 37 °C.

### Reprogramming experiments

ColSC/M2rtTA/Oct4-GFP cells were provided by Professor Ihor Lemischka (Mount Sinai School of Medicine, NY). Cells were expanded from P0 to P2 and Thy1^+^ cells were FACS sorted (see FACS section) to start with an homogeneous cell population. Thy1^+^ cells were cultured in 35 mm plates pre-coated with 0.1% gelatine for at least 30 min at a density of 2 × 10^5^ cells/well. MEFs were grown in 2 ml reprogramming media containing DMEM with 15% FBS (Gibco, USA), 2 mM L-glutamine (Sigma, USA), 100 U/ml penicillin and streptomycin, 0.1 mM NEAA (Gibco, USA), 0.05 mM β-mercaptoethanol (Sigma, USA), 1000 U/ml Leukaemia inhibitory factor (LIF) and 1 μg/ml doxycycline (Sigma, USA) at 5% CO_2_ and 37 °C^4^. Reprogramming media was changed every day during the reprogramming time course. Doxycycline was removed from media on day 12 and cells were cultured in doxycycline-free media for another week (day 19). Cells were not passaged at any time during the course of a reprogramming experiment^20^. Reprogramming experiments were performed in biological triplicates.

### FACS

For generation of spectral library reprogrammed Oct-4^+^ cells (iPSCs) were labelled with Fixable Viability Dye eFluor® 450 (eBiosciences, USA) and isolated based on Oct4-GFP expression at the Flow Cytometry Facility (Cancer Research UK Manchester Institute, Manchester). Cells were sorted using a BD Influx Cell Sorter (BD Biosciences; BD FACS^TM^ Sortware sorter software).

For SWATH-MS, cells were harvested by incubation with TypLE on days 3, 6, 10 and 13 reprogramming, following treatment with doxycycline. Cell suspensions were labelled with Fixable Viability Dye eFluor® 450 (eBiosciences, USA) to exclude dead cells and debris. Cells were incubated with PE-conjugated rat anti-Thy1 (Life Technologies, USA) and Alexa Fluor 647 conjugated mouse anti-SSEA1 antibodies and sorted using a BD Influx Cell Sorter (BD Biosciences; BD FACS^TM^ Sortware sorter software) and Aria II and III systems (both BD Biosciences; FACSDIVA^TM^ software). Corresponding isotype controls were used as negative controls for gating purposes. The following populations were collected on each day of the reprogramming time course: Thy1^+^ and Thy1^−^ populations on day 3; Thy1^+^, Thy1^−^ and SSEA1^+^ populations on days 6 and 10; and Thy1^+^, Thy1^−^, SSEA1^+^/Oct-4^−^ and SSEA1^+^/Oct-4^+^ cells on day 13 and 20.

### Sample preparation

Cells were lysed with 0.5 M TEAB, 0.05% SDS, 6.4 mM sodium pyrophosphate and 1 mM sodium orthovanadate (Sigma, USA). Lysates were sonicated using a Misonix Xl2020 sonicator Ultrasonic Processor for cell disruption. Cell debris was removed by centrifugation and prior to the proteolytic digestion. Protein samples were reduced with 5 mM dithiothreitol (DTT), carbamidomethylated with 15 mM iodoacetamide and overnight digested with trypsin (estimated enzyme/protein ratio of 1:20; Promega, USA). Trypsin was inactivated by lowering pH to 2 with formic acid. For spectral library generation 500 µg of protein were digested and peptides were cleaned-up using SepPak C18 cartridges (Waters, USA). Peptides were washed five times and eluted (80% acetonitrile and 0.1% FA) and solvents were evaporated in a vacuum centrifuge. For SWATH-MS acquisition, 10 µg of protein were digested and cleaned-up using strong anionic exchange resin beads (POROS 50 HG Strong anion Exchange Resin; Thermofisher Scientific, USA).

### High pH fractionation of peptides for spectral library generation

500 µg of four peptide samples including NIH3T3 (MEFs), ColSC/M2rtTA/Oct4-GFP cells on day 6 of reprogramming, FACS sorted iPSCs (ColSC/M2rtTA/Oct4-GFP Oct-4^+^ cells) and iPSCs (ColSC/M2rtTA/Oct4-GFP Oct-4^+^ cells) and feeders were fractionated using an Agilent ZORBAX Extend 80 Å C18, 4.6 × 150 mm, 3.5 µm on an Agilent 1100 HPLC (Santa Clara, CA, USA) operating at 1 ml/min. Mobile phase A consisted of 5% acetonitrile (v/v) with 0.1% ammonium hydroxide (v/v) and mobile phase B consisted of 95% acetonitrile with 0.1% ammonium hydroxide. Both buffers were adjusted to pH 10 with ammonium hydroxide. Samples were injected onto the column at 1 ml/min for 60 min after which peptides were eluted in a 25 min gradient from 3 to 27% B. Ninety fractions were collected per sample and combined into 30 post-concatenation fractions from early, middle and late stage fractions and lyophilised. Peptides were reconstituted with 3% (v/v) acetonitrile and 0.1% (v/v) formic acid in water with iRT peptides (Biognosys, Switzerland; PN: Ki-3002) following the manufacturer’s instructions^23^ prior the injection into the mass spectrometer.

### DDA acquisition of samples

Peptides were injected directly into a TripleTOF 6600 (AB Sciex, Warrington, UK) coupled to an Eksigent Nano LC systems (Eksigent, Dublin, CA). Mobile phase A consisted 3% acetonitrile and 0.1% formic acid in water and mobile phase B 97% acetonitrile and 0.1% formic acid in water. Peptides were separated with a 300 nl/min linear gradient of 3–30% B for 90 min, 30–40% B for 10 min, 80% B for 5 min and 3 min equilibration at 3% B. The 30 most abundant ions were selected for MS/MS following a 250 ms TOF-MS survey scan and 50 ms MS/MS scan. Dynamic exclusion time was set to 15 s. Selected parent ions had charged states between +2 and +5 and were fragmented by collision-induced dissociation (CID). Experimental study information is summarised in supplementary Table 1.

### Data re-use

Raw data from a previously published spectral library derived from mouse tissues^12^ was downloaded from Pride to complement the cell derived data collected above. Data was downloaded from Pride repository PXD002896 and processed through the same pipeline detailed below as the newly acquired data. In this way, a spectral library with unified annotation and quality was generated from newly acquired and complementary historic data.

### Spectral library generation

The workflow for spectral library generation was scripted using gnu-make in line with the previously published workflow^13^ with some modification. The pipeline was scripted and both script and detailed accompanying documentation are included with raw data in the Pride repository, see section Code Availability below. The documentation describes in detail the workflow managed by the gnu-make script including every command line used to process raw data to finished library. In brief, the process is as follows: DDA data in proprietary format (.wiff/.wiff.scan for Sciex, .raw for thermo) data was converted to centroided mzxml format using proteowizard version 3.0.20002. These spectra files were then searched against a protein database combining the canonical mouse proteome (UP000000589) from the EBI (https://www.uniprot.org/proteomes/UP000000589) including both reviewed and unreviewed sequences; the iRT protein sequence; common contaminants and a locally generated decoy database using X!Tandem v2017.2.1.4 search engine with the following parameters: parent mass error ±30 ppm, product mass error ±25 ppm, carbamidomethylation on cysteine as fixed modification and oxidation on methionine as variable modification, for complete details see X!Tandem setting files incuded with documented make script. Search results were combined using transproteomics pipeline v5.2.0 tools xinteract and peptideprophetparser. Peptide score required to give a FDR < 1% was found using Mayu^24^ and spectra meeting this criteria were imported into spectrast v5.0 using iRT normalisation and excluding decoy proteins and contaminants. Subsequently consensus libraries were produced for each cell and tissue type, and also combined into further consensus libraries for all cell types, and all cells and tissue combined. A further library was generated by re-mapping against the reviewed (swiss-prot) protein sequences. The last two libraries are likely to be the most widely re-used since it has the largest coverage of the proteome. Libraries in spectrast.sptxt format were further converted into tab separated format using the spectrast2tsv python script from msproteomicstools v0.8.0 and then to .TraML format with TargetedFileConverter from openMS v 2.4.0. The resulting libraries are therefore available in formats for several DIA analysis tools: The spectrast .sptxt format can be opened directly in Skyline; the .TraML format may be used with openSWATH; with a small alteration to the spectrast2tsv command detailed in the documentation a library suitable for import into Sciex Peakview software may be generated. These last steps are application specific and users will need to adjust parameters to ensure they are optimal for intended use.

### Spectral library validation with SWATH data and Skyline

FACS sorted reprogramming populations were lysed, proteins digested (as above) and samples were acquired using the same TripleTOF 6600 (AB Sciex, Warrington, UK) and Eksigent Nano LC systems (Eksigent, Dublin, CA) for SWATH data acquisition. This was performed in biological triplicates. Day 20 SSEA1^+^/Oct-4^+^ cells used for quantitative validation of spectral library were also run in technical replicates.

Peptides were separated by reverse-phase chromatography at a flow rate of 300 nl/min. Protein digest were injected onto a pre-column (waters C18, 100 Å pore size, 5 µm particle size, 180 µm × 20 mm internal diameter) and analytical column (Waters C18 1.7 µm particle size, 130 Å pore size and 75 µm × 250 µm internal diameter). Gradient was formulated with mobile phase A containing 3% (v/v) acetonitrile with 0.1% (v/v) formic acid and mobile phase B containing 97% (v/v) acetonitrile in 0.1% (v/v) formic acid. Peptides were separated with a linear gradient of 3% to 30% B for 90 min, 30% to 40% for 10 min, ramped up to 80% B in 5 min. Gradient was held at 80% B for 5 min and column was equilibrated at 3% B for 3 min.

Peptides were injected directly into a TripleTOF 6600 operated in product ion scan mode. A hundred overlapping windows (1 Da overlap) of variable width ranging between 6 and 50 Da covered a mass range of 400–1250 Da. Collision energy was different for each window. SWATH-MS accumulation time was set to 25 ms for each fragment ion scan and 250 ms for the survey scan with a total cycle time of 2.75 s. Single injections of biological triplicates were performed. Experimental study information is summarised in supplementary Table 1. Window size and collision energy information for each SWATH experiment is described in supplementary Table 2.

The combined iPSC and tissue library was opened in skyline v20.1.0.76. The iRT peptides sequences were imported. A new retention time database was then created with the iRT standards and the spectral library imported above added. All the raw SWATH data was then imported and a retention time calibration based on the iRT peptides was calculated. Skyline was then set up to filter peptide sequences and import data. In “Peptide Settings” under “Digestion” the enzyme was set to trypsin [KR|P] and a background proteome was set up based on the same fasta file against which the library was generated. Under “Filter” skyline was set to accept only peptides 8–18 amino acids long; exclude 5 N-terminal amino acids; exclude peptides contacting cysteine, methionine, and arginine-proline and lysine-proline pairs. In “Transition Settings” under “Filter” skyline was set to accept precursor charges 2 or 3; ion charges 1 or 2 and ion types y and b. Product ion selection was set to m/z > precursor to 6 ions with ion N-terminal to proline as special ion. Tick boxes to use DIA precursor window for exclusion and auto-select all matching transition were selected. Under “Full Scan” MS/MS filtering the acquisition method was set to DIA, the product mass analyser was set to TOF, the isolation scheme was programmed in as outlined above and the resolving power set to 20,000. Retention time filtering was set to use scans within 7 minutes of the predicted iRT. The tick box for high-selectivity extraction was left unticked. The spectral library exploration window was then opened and all the peptides in the library imported and associated with proteins by reference to the Uniprot mouse proteome against which the spectral library was generated. Decoy peptides were then generated against the imported peptides by random mass shift. All data was then imported into the skyline document. An mProphet peak scoring model was trained against the decoy and second best peaks using score parameters: Intensity; Library intensity dot-product; Shape (weighted); Co-elution (weighted); co-elution count; signal to noise; and product mass error. The retention time difference score did not make a significant contribution and was excluded. Peptides without a match given a *q*-value of less than 0.01 in at least one data set were then excluded from the analysis document. A new set of decoy peptides were generated against the reduced set of peptides and the model re-trained to give a refined set of *q*-values. All peaks were re-integrated using the newly trained model. Data tables with retention time and spectral data were exported from skyline for further analysis in R. The Skyline document and all associated files are included with the data deposition in PRIDE.

#### Statistical analysis

Tabulated spectral libraries and skyline data exports were imported into R v3.6.2 for analysis. Spectral similarity was calculated using the SpectrumSimilarity function from the OrgMassSpecR package. The proportional Venn diagram was generated using the nVennR package. All other graphics were generated using the ggplot2 package. A documented script describing processing of the data and generation of the figures is included in the data deposition in PRIDE.

## Data Records

The data submission^17^ includes six consensus libraries, one for each of the four cell samples: NIH3T3 (MEFs), ColSC/M2rtTA/Oct4-GFP cells on day 6 of reprogramming, FACS sorted iPSCs (ColSC/M2rtTA/Oct4-GFP Oct-4^+^ cells), ‘iPSCs (ColSC/M2rtTA/Oct4-GFP Oct-4^+^ cells) and feeders, one for reprocessed tissue data and a consensus library for all five individual libraries. The libraries are included in .sptxt, .traml, and both openms and peakview .tsv formats to facilitate re-use. The most useful files are likely to be the set with base names: splib_all_ipsc_Tissues_Cons_SP with suffixes as above. These include all the cell and tissue derived spectra and are mapped against the non-redundent swissprot protein database.

The raw mass spectrometry data and key intermediate results are also included in the deposition. The raw cell sample data in Sciex .wiff/.wiff.scan format and the tissue data in thermo .raw format. Processed spectra are included as .mzXML, search results are included as both .pep.xml and .mzid format. The two example SWATH files and the skyline analysis are also included as demonstration of the library’s application.

There is also a documented gnu-make script included which should enable libraries to be completely re-built from the raw data in the submission.

## Technical Validation

### Control of false discovery rate

**Fig. 1:**
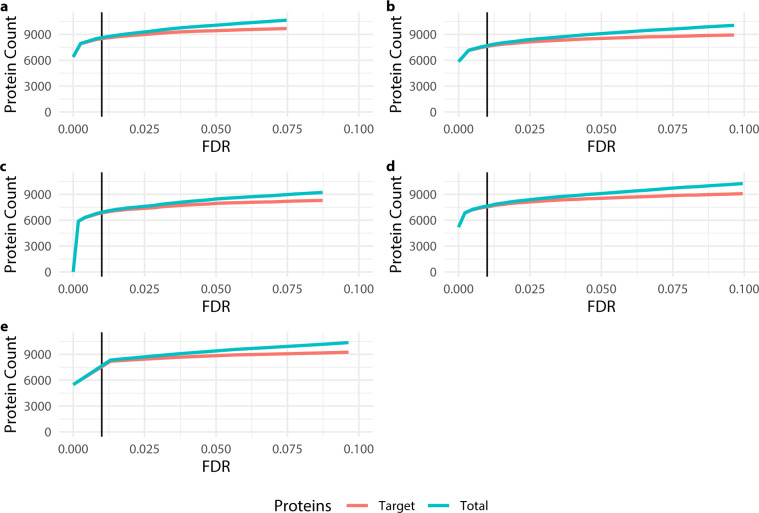
Plots of the target and total protein count for each sub-library. (**a**) MEF, (**b**) Day six of MEF-iPSC transition, (**c**) IPSC and feeder cells (MEFs) mixed, (**d**) iPSC and (**e**) tissue data sets. In each case the vertical line shows the chosen FDR threshold.

The principle criteria for quality in a spectral library for bottom up proteomics is the false discovery rate (FDR). This is because false peptide-spectrum matches (PSM) are annotated with incorrect retention times and product ions. A spectral library populated with such spectra therefore threatens future analysis based on the library. Here the MAYU^24^ strategy was used to control the protein false discovery rate at 1% as is typical for similar libraries reported else where^9,13^. The assay saturation curves reported for each of the four cell types and tissues (Fig. 1), show that this stringent protein cut-off achieves near assay saturation for this data set.

### Coverage of the mouse proteome proteins and peptides per protein

The library is available mapped against both complete and the reviewed subset of the EBI mouse proteome.

**Fig. 2:**
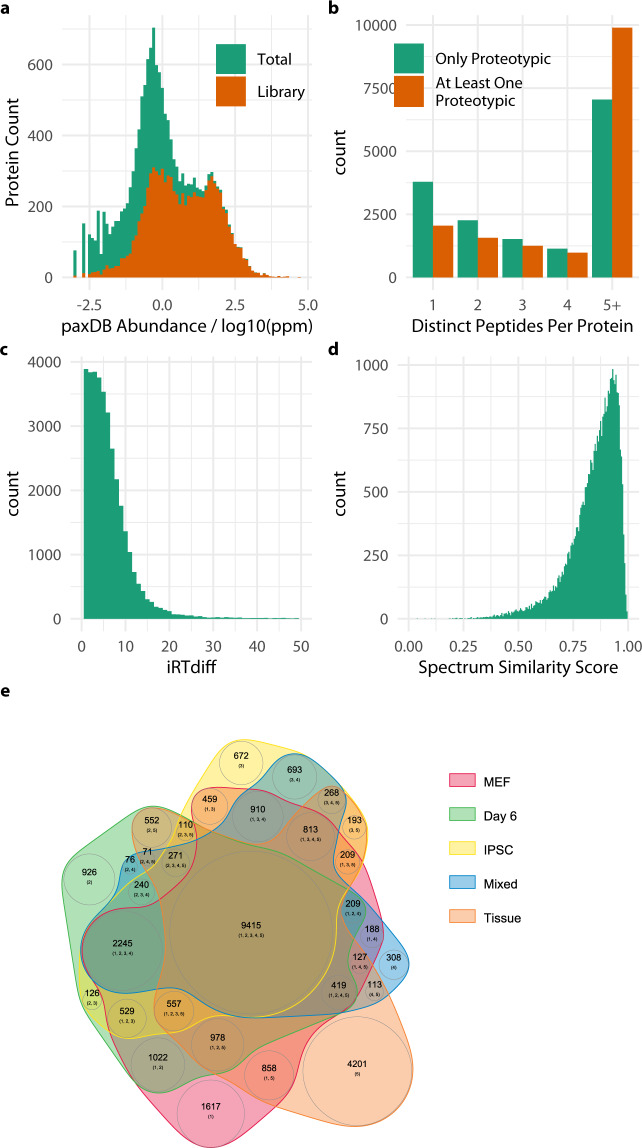
Plots showing quality of libraries after filtering to Swissprot protein entries. (**a**) Histogram of mouse protein abundance from paxDB database with proteins identified by proteotypic peptides in the final library overlayed. The library covers almost all of the most abundant proteins in the mouse proteome. (**b**) The numbers of proteins identified by 1–4 and 5 or more proteotypic peptides. (**c**) The difference in iRT score between peptides identified in both cell culture samples and re-analysed tissue samples. (**d**) Distribution of similarity scores (cosine of matched signal intensity vectors) between cell culture and tissue derived data. (**e**) Venn diagram for numbers of proteins uniquely identified across all combination of sample sets.

The complete mouse protein database used in this study contained 55408 entries. Of these the total number of proteins identified was 29519 (53%), however the number of those identified by proteotypic peptides was 7435 (13%). The depth of coverage by abundance was investigated by taking the estimated mouse whole organism protein abundance from paxDB (https://pax-db.org/) and plotting the histogram of those proteins identified by proteotypic peptide over the whole pax-db dataset, which itself covers 89% of the mouse proteome (Fig. 2a). The majority of the more abundant proteins in the mouse proteome are represented in the spectral library although lower abundance proteins remain under represented. It is doubtful that these low abundance proteins would be accessible to SWATH analysis without specialised and targeted sample preparation.

The reviewed (swiss-prot) proteome comprises 17048 entries. Of these the spectral library mapped to that proteome identifies 10148 (60%) proteins, however the number identified by prototypic peptides was 9871 (58%).

### Peptides Per Protein

The spectral library is comprised of peptide-spectra-matches. After matching the peptides are grouped into the proteins from which they are derived by the tryptic digest. Some of these peptides are unique to a single protein, so that when quantified they uniquely identify and quantify that protein, these are termed proteotypic peptides. Some peptides are shared across multiple proteins either because of conserved sequences between genes or single genes coding for multiple proteins. Thus proteotypic peptides are the most specific tools to quantify a single protein. Shared peptides may also be valuable where prototypic peptides are un available, in situations where only one of the theoretical proteins sharing the peptide is expressed, or where shared between similar proteins they quantify classes of proteins. The use to which they are put will depend on the research question.

Considering only prototypic peptides 7435 proteins were represented by at least one, 1813 by only one and 5622 by two or more distinct peptide-spectra matches. It is quite common to supplement a protein identification and/or quantification supported by a single proteotypic peptide with additional matches to shared peptides. When shared peptides are considered for proteins identified by at least one proteotypic peptide the number of proteins identified by only one peptide fell to 640, and by two or more rose to 6795 (Fig. 2b). Considering the full set of 29376 proteins and all the peptides associated with them 5592 proteins were identified by a single and 23784 by two or more peptides.

### Validation of iRT Attribution

Libraries were constructed from two separate sources of raw data, the locally generated cell culture data on the Sciex eksigent-6600 QTOF system and the tissue data on the Thermo EASY-nLC-Q Exactive Plus system^12^. This provided the opportunity to compare the iRT scores for each data set to assess how comparable the datasets were. The iRT score of 52% of peptides differed by less than 5 and 85% by less than 10 (Fig. 2c).

### Validation of spectra

In a similar way to the comparison of iRT scores, it is possible to compare spectra for shared spectra between spectral libraries with differing sources. Pairs of spectra with matching peptide annotations between the historic tissue library acquired on Thermo systems and the current cell culture library acquired on Sciex systems were scored by cos theta of the angle between intensity vectors representing the annotated peaks in the spectra. The score of 90% of spectra was higher than 0.7, which indicates highly similar spectra. A brief investigation of pairs of spectra with low similarity scores indicated severe attenuation of either low or high mass signals in the differing spectra. This may indicate erroneous peptide spectral matches or differing behaviour between instruments.

### Complementarity with tissue data and coverage of fibroblast to iPSC transition

A total of 9446 proteins were identified in all four cell and the tissue libraries. Of the proteins uniquely identified in each data set of the iPSC reprogramming time course 1622 were identified only in MEF cells, 926 at day 6 and 673 iPSC, this indicates sampling across the time course covered more proteins potentially involved in or caused by the reprograming process. The mixed sample containing iPSCs and feeder cells (mitotically inactive MEFs) uniquely identified 308 proteins. This is the lowest number of uniquely identified proteins, which makes sense, as the sample was an average of the others. A further 4237 proteins were uniquely identified in the tissues data set demonstrating the value of integrating data from differencing sources to generate a deeper spectral library.

### Inclusion of iPSC markers in library

All of the desired markers for study of iPSC reprogramming are represented in the library including fibroblast markers Col5a2, Fibrillin2, Thy1 (Uniprot IDs: Q3U962, Q61555 and P01831) and iPSC markers Oct-4, Nanog, Esrrb and Fbx15 (Uniprot IDs: P20263, Q80Z64, Q61539 and Q9QZN0).

### Comparison with other murine libraries

Two previous murine spectral libraries have been published (Zhong *et al*.^10^, Krasney *et al*.^11^). Both these libraries incorporate both murine tissue and cell lines and were mapped to the reviewed Uniprot library, they are compared to the Swissprot mapped version of our library. The Zhong library contains spectra from the iPSC marker Fbxo15 whilst the Krasney library contains none of the iPSC markers. This is to be expected since neither of these libraries focused on the iPSC reprograming. A further 462 proteins are unique to our library and may also be involved in the reprogramming process. Thus the library present here is uniquely applicable to iPSC reprograming in mouse cells.

### Example application of spectral library to SWATH data

**Fig. 3:**
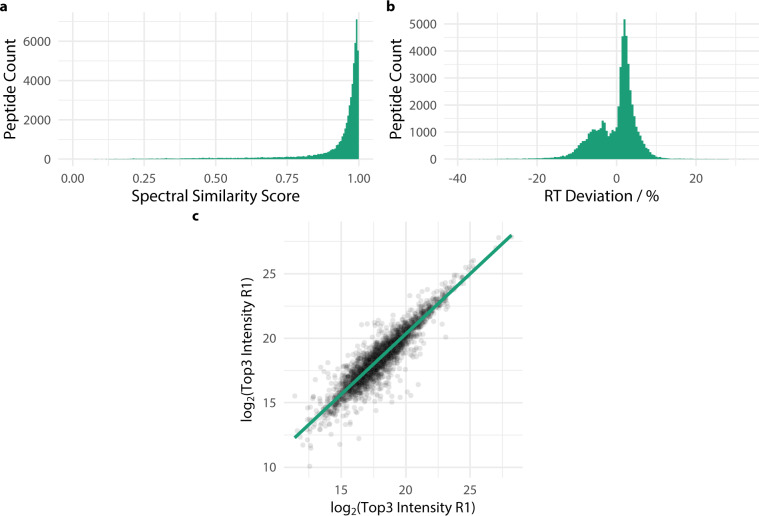
Plots showing applicability of library to SWATH analysis. (**a**) A histogram of spectral similarity scores between library spectra and SWATH analysis transition relative intensity. (**b**) percentage deviation between libraries predicted and measured retention time. (**c**) Plot of log2 transformed sum of “top 3” protein quantification from a pair of injections plotted against each other. A linear model through the data is plotted over the data.

Utility of the spectral library for SWATH analysis was demonstrated by applying the library to analysis of samples collected over a time course of reprogramming iPSC cells. Across the whole analysis, 2015 proteins were quantified at a peptide FDR of 1%. The comparability of spectra generated for the library by DDA analysis and the relative intensities of transitions identify in the SWATH in the same way as spectra from differing sources were compared above (Fig. 3a) again 91% of spectra had a score greater than 0.7 indicated close agreement between library spectra and the relative intensity of SWATH transitions. There was also close agreement between retention time as predicted in the library and returned by the skyline analysis (Fig. 3b) with 95% of identified peptides within 10% and 99% within 20% of the expected retention time. The apparent bimodal distribution appears to arise from a disparity in predicted early (<40 min on a 2 hour gradient) retention times from the tissue data which was acquired on entirely different system from the cell data which was acquired on the same system as the SWATH. That disparity disappears at later retention times.

To further validate the library a pair of technical replicate injections of a single iPSC sample (SSEA1^+^/Oct-4^+^ cells on day 20) were processed through skyline against the complete library. At a q-value of 0.05 9493 peptides comprising 3229 proteins were quantified. Peptides were quantified by the log2- transformed total area of fragments and gave a Pearson correlation of 0.881 (p-value < 2.2e-16 95% CI 0.877–0.885). Proteins were quantified by the “top three” method in which the log2-transformed total area of fragments for up to the top three highest signal peptides were summed, this gave a Pearson correlation of 0.922 (p-value < 2.2e-16 95% CI 0.917–0.927). A scatter plot of this data shows the tight dispersion and linear relationship of the results (Fig. 3c).

The use case for the data was to investigate the production of iPSC cells which required iPSC markers be quantified by SWATH analysis. Two of the three fibroblast markers (Col5a2 and Thy1) were quantified in the fibroblast samples and two of the four iPSC markers (Oct-4 and Fbx15) were quantified in the iPSC samples. The capability to identify these particular proteins relevant to the specific application for which the data set was intended, combined with the other encouraging metrics presented for the library confirm its value as a shared community resource. The library will contribute to study of mouse and mouse cell samples and also to the study of reprogramming of iPSC from fibroblasts.

## Usage Notes

The spectral libraries are presented with just the mouse spectra and without decoy or contaminant spectra. This enables those re-using the libraries to subset the library, append appropriate contaminant libraries and generate decoy spectra as appropriate for their use case and processing pipeline. All proteins are identified by their Uniprot accession number which is widely used and can readily be converted to other protein or gene naming schema using online tools (https://www.uniprot.org/uploadlists/).

Complete libraries are presented as .splib/sptxt format for spectrast, the .TraML standard format, .tsv formats for both openSWATH and Sciex SWATH 2.0 based pipelines and .blib format for skyline. This ensures easy re-use of the data on all common SWATH analysis pipelines. Modification of .tsv formats to accommodate different swath window schema may be required and is documented with the script.

Processing of raw data into a spectral library is fully scripted and documented enabling complete reproduction of library generation process from raw data to library. The process is closely modelled on a previously published process^13^. This will enable easy re-production of libraries with updated software tools or sequence databases as they become available. The script may also form the basis for variant processing pipelines applicable to particular use cases by replacing tools in the pipeline or building the library against alternative protein sequence libraries.

The pipeline also demonstrates the combination of data sets acquired on separate systems by separate groups. It may form a template to include yet further spectral data to expand coverage of the mouse proteome, or for re-use as a general library supplementing a project specific library covering proteins of particular interest.

## Supplementary information

Supplementary File 1

Supplementary table 1

Supplementary table 2

## Data Availability

The workflow for spectral library generation was scripted using a gnu-make. The make file and a companion document are included with the data in the pride repository^17^, the make file companion document is also included with this article as supplementary file 1. The make file and the companion document are available on github (https://github.com/M-Russell/Mouse_iPSC_Spectral_Library). These files should enable precise replication of the library from raw data as presented here and re-use of the raw data through varied processing. The library was created with a series of open source software packages, the precise versions and sources of these programs are given in the documentation. Python scripts are required for the pipeline and instructions are given on how to install the versions used.
