# Supplementary material for: Generation of a mouse SWATH-MS spectral library to quantify 10148 proteins involved in cell reprogramming
Source: Sci Data. 2021 Apr 26;8:118. doi: 10.1038/s41597-021-00896-w (PMC8076245; doi:10.1038/s41597-021-00896-w)
Supplement: Supplementary file 1 [file 41597_2021_896_MOESM1_ESM.pdf]

# Documented Mouse Spectral Library Producing Makefile

Matthew Russell

October, 2020

**Abstract**

Documentation of a makefile to reproduce spectral libraries derived from a time course of mouse cells reprograming to pluripotent stem cells. A complete comentry on the accompnying makefile script to enable reproduction of libraries from raw mass spectrometry data files.

## Contents

**Introduction** **2**

**Prepare System For Script** **2**

Install All Required Programs . . . . . 2
Prepare Directory Tree For Library Generation . . . . . 3

**Run Make on Makefile** **5**

Execute Make . . . . . 5
After Complete Library Build . . . . . 5

**Documented Makefile** **9**

Specify the Shell . . . . . 9
Set Shell Environment . . . . . 9
Set Explicit Paths to Executables . . . . . 10
Raw Data Files . . . . . 10
Protein Sequence Database (.fasta) Files . . . . . 13
Lists for Target and Intermediate Files . . . . . 13
Make All Targets . . . . . 15
Convert Raw Mass Spectrometry Data to mzXML . . . . . 15
Combine Protein Sequence Databases and Add Decoys . . . . . 17
X!Tandem Search . . . . . 18
Converting `.t.xml` Fileto `.mzid` For Upload to Pride . . . . . 18
Combine Search Results and Process Through Trans-Proteomics Pipeline . . . . . 19
Controlling False Positive Rate . . . . . 21
Building Individual Libraries with Spectrast . . . . . 21
Combine Libraries . . . . . 23
Remap Combined Library to SwissProt Libary . . . . . 24
Conversion of Library into Other Formats . . . . . 24
Additional Input Files . . . . . 26

**Conclusion** **34**

**References** **34**

# Introduction

This document provides instructions for the reproduction of spectral libraries reported in [paper reference] from the raw data held in Pride repository [PXD017014].

The document facilitate re-use of the date by making the processing pipeline entirly reproducable through running the makefile. It should support users to implement the pipeline on their own system. There are links throughout to the supporting documenation for each tool used which should support bug fixing in a new setting, and notes on issues encountered constructing the pipeline that should help avoid bugs. The detail in the document should enabel modification to the data processing pipeline. Possible modifications include adjusting “swath window” schema to acomodate spesific experiments and swapping out software tools perfroming spesific steps in the analysis for future versions or entirly alternate tools. The details documentation also enables scrutany of the current pipeline and the choices made in its construction.

The libraries are built using a script for the build automation tool gnu-make v4.2. The script, termed a “makefile”, passes a set of comand line recipies to make instructing it how to convert raw data through intermediate file types to the desired final product. A high level description of `make` can be found on its Wikipedia page, comprehensive documentation is available on its manual page. The makefile is extensively documented below explaining key concepts and indicating places where manual editing may be required.

# Prepare System For Script

The script was developed on the Windows 7 64 bit service pack 1 operating system and subsequently re-run on windows 10 v1909 OS build 18363.592 to produce the submitted data. The script must be run on windows rather than a unix system to use the `qtofpeakpicker` program from proteowizard which extracts spectra from 6600 qtof data. Several GNU programs are used in addition to bioinformatics scripts, links to sources for all the programs are included in the instructions below.

## Install All Required Programs

(1) If not present on the system download and install `gnu-make v4.2.1` this is best installed as part of mingw-w64. The installation process offers multiple versions and sub-variants. For this work the installation was: version: 8.1.0; Arch: x86_64; Threads: win32; exception: seh; build revision: 0. Several versions of `make` were tested during development of this script. The version of make that comes with `Rtools` used as the source of `sed` and `gawk` below did not work on our system. The compiled version for windows (`gnu-make v3.81`) available from GNU is also not compatable.

(2) If not present on the system download and install `sed v4.4`. This is included with Rtools used here but any of the several `sed` distributions for windows should work.

(3) If not present on the system download and install `gawk v4.2`. This is included with Rtools used here but any of the several `gawk` or `awk` distributions for windows should work.

(4) If not present on the system download and install proteowizard, this work was done with v3.0.20002.36ad78f14.

(5) If not present on the system download and install the Trans Proteomic Pipeline instructions are available, this work was done with v5.2.0. During install select custom installation and install `Apache web server` and `Strawberry Perl`.

(6) If not present on the system download and install the X!Tandem search engine, this work was done with version Alanine (2017.2.1.4).

(7) If `mzid` files are required and not present on the system download and install the latest 64 bit java release.

(8) If mzid files required and not present on the system download mzidlib unzip the resulting file and place in an accessble location. This work was tested with v1.7.86.

(9) If not present on the system download and install openMS this work was tested with v2.4.0.

(10) A python distribution is required to run the msproteomicstools package. This work was tested with miniconda for python 3.7 with msproteomicstools installed as per the instructions given on the website. The minimal python installation enables just the requirements for this process

to be installed with the versions required to replicate this work. Miniconda may be installed alongside other instances of python allowing this work to be repeated without disrupting a pre-exisiting system. During installation note the target directory. For single user installation this will look like: `C:\Users\<user>\AppData\Local\Continuum\miniconda3`, for all users it may be `C:\ProgramData\Miniconda3`. Note `C:\ProgramData\` is a hidden directory on windows 10, navigate to it directly by entering path into windows explorer or set windows explorer to show hidden files.

(11) Install requred python packages. To access the python comand line, open a windows comand window; navigate to the `Miniconda3\Scripts` directory; and type `activate` into the comand line. Required packages will need to be installed from `pipy.org` through the python comand line. The following comands entered into the anaconda prompt should install the required packages in the versions used here. Alternativly the latest versions of these programs might be installed although that might require adjustments to processing comands below.

```
pip install numpy==1.15.3
pip install pymzml==0.7.5
pip install Biopython==1.72
pip install Cython==0.29.2 --install-option="--no-cython-compile"
pip install msproteomicstools==0.8.0
```

No further interation with the python environment should be required.

(12) Optionally install `R`, `Rstudio` and `texlive` to process this file: `makefileDoc_WindowsPClibrary.Rmd` through `knitr` to output the makefile and additional input files. If you do not know what this means it is probably simpler to download the required files from the Pride repository.
(13) An FTP client is useful for downloading data from the EBI Pride Archive. Alternative downloading tools exist. This work was tested with WinSCP v5.13.3.
(14) A command line terminal is required to initialise and monitor the `make`. Microsoft windows' `cmd.exe` is sufficient, see below for instructions. However we recommend an alternative interface called MobaXterm which is marketed as a user friendly toolbox for remote computing but which also runs the windows comand line. MobaXterm is more legible than `cmd.exe` which makes debugging and running scripts easier. Obtain MobaXterm by downloading from the link and either install or use the portable edition. Another alternative terminal is windows-terminal available from the Microsoft Store.

If microsoft windows `cmd.exe` is to be used access the program by clicking on the start symbol; type `cmd` into the search box; the top search result will be the `cmd.exe` and should be selected; hit return or click on the `cmd` program to open the comand window. To increase the width of the window right click on the frame and select properties; in the `layout` tab increase the `screen buffer size` width and `window size` width untill the window fills the screen. It will also be helpful to enable `QuickEdit Mode` and `Insert Mode` in the `Options` tab.

## Prepare Directory Tree For Library Generation

The following describes how to set up a the directroy structure to build all the libraries:

### Prepare the Input Files.

(15) Select or create a base directory in which to build the library.
(16) Within this directory create a directory called `ini`.
(17) Within this directory create a dir called `PXD002896`
(18) Within this directory create a dir called `PXD017014`
(19) Download the mouse proteome UP000000589 from the Uniprot website. To do so, follow the link, then click on the download button over the list of chromosomes. Ensure the `Download all protein entries` radio button is selected; the format is set to `FASTA (canonical)` and the final radio button `compressed` is selected. Click `Go`. A compressed .fasta.gz file will be downloaded. Extract this file and move it to the directory created in (15). Remove the `%` from the file name as this character has special meaning for `make` and will cause errors.

(20) Download the common contaminants database crap.fasta from the GPM.
(21) Download the common contaminants database contaminants.fasta from the Max Planck Institute of Biochemistry.
(22) **Either:** Copy this file `makefileDoc_WindowsPClibrary.Rmd` to the base directory with the addition of files `nature.csl` and `library.bib` downloaded from Pride repository `PXD017014`, open in Rstudio and "knit" to html or pdf. This will write the required additinal input file to the `ini` directory and create the required makefile in .
**Or:** Download files: `default_input.xml`; `irtfusion.fasta`; `iRTPeptides.txt`; `SWATH_windows.txt`; `taxonomy.xml` and `Xtandem_params.xml` from Pride repository `PXD017014` to directory "ini" and file `iPSCwindowSpecLib.mk` to the base directory. See below for FTP download instructions for winSCP if required.

### Download Raw Data and Script

(23) Open WinSCP. Click on the `New Session` tab, a login screen will appear; click `New Site` and enter `File Protocol`: FTP, `Host Name`: `ftp.pride.ebi.ac.uk/pride/data/archive/2015/11/PXD002896`, and click the `Anonymous login` box.
(24) Download all `.raw` ms data files to directory `<baseDir>\PXD002896` created in (17).
(25) Return to WinSCP. Click on the `New Session` tab, a login screen will appear; click `New Site` and enter `File Protocol`: FTP, `Host Name`: `ftp.pride.ebi.ac.uk/pride/data/archive/2020/01/PXD017014`, and click the `Anonymous login` box.
(26) Download all `.wiff` and `.wiff.scan` ms data files to directory `<baseDir>\PXD017014` created in (18).
(27) Download file `iPSCwindowSpecLib.mk` to the `<baseDir>` created in (15).

The directry structure should now appear as follows. Pride repository directories are illustrated containing example raw data files.

```
<baseDir>
  +----ini
        +---uniprot-proteome3AUP000000589.fasta
        +---crap.fasta
        +---contaminants.fasta
        +---default_input.xml
        +---irtfusion.fasta
        +---iRTPeptides.txt
        +---pepCalPeptides.txt
  +----PXD002896
          +---ErikM_S1406_034.raw
          +---ErikM_S1406_035.raw
          +---...
  +----PXD017014
          +---BAND_1.wiff
          +---BAND_1.wiff.scan
          +---...
  +----iPSCwindowSpecLib.mk
```

(28) Prior to execution the makefile `iPSCwindowSpecLib.mk` requires editing to ensure it is correct for the environment and indended application. In particular paths to executables must be set correctly and swath windows adjusted to match the intended aquisition method. See the documentation for the makefile below for details.

# Run Make on Makefile

## Execute Make

(29) Once all the files are downloaded into the required directories and the makefile `iPSCwindowSpecLib.mk` has been edited for the system on which it will be executed, see below, `make` may be invoked on the edited makefile as:

```
# Set the path to the location of mingw32-make.exe or version of make required.
# Note that other sources of make may be used see above.
PATH="C:\Program Files\mingw-w64\x86_64-8.1.0-win32-seh-rt_v6-rev0\mingw64\bin"

# Run make on the iPSCwindowSpecLib.mk file.
mingw32-make.exe -rR -j 8 --output-sync -f iPSCwindowSpecLib.mk
```

The switches chosen for `make` are as follows:

**-rR** Ignores the internal rules for compiling `C` programs, this simplifies debuging information
**-j n** Allows make to submit upto n concurrent jobs, eight in case of the example code. The this should be adjusted for the system used to build the libraries, if the system is dedicated to the process one job fewer than the total logical cores available works well.
**--output-sync** Outputs all the console information after completion of a recipe. This prevents the confusion of several verbose tools such as x!tandem or spectrast writing out interwoven and garbled output as they run.
**-f iPSCwindowSpecLib.mk** Indicates the makefile which defines the building pipeline.

An additional switch that may be useful whilst debugging the script include:

**-W** Which sets files to assume are infinitly new. Thus `-W *.mzxml` will assume all .mzxml files are new, and will perform all searches etc after converstion to `.mzxml` but will not repeat the converstion; `-W *.pep.xml` will skip the search process and and move on to library generation steps. This procedure can be applied top any part of the workflow.
**-n** Prints the recipies to update files without actually executing them.
**-B** Re-builds all files.

Full documentation of all options can be found on the manual website.

## After Complete Library Build

After the completion of the library buidling process the directroy created in (15) above will contain a further directory `lib` into which files are organised as follows:

```
<baseDir>
    +----lib
            +---all_ipsc
            |       spectrast.log
            |       splib_all_ipsc_Cons.pepidx
            |       splib_all_ipsc_Cons.spidx
            |       splib_all_ipsc_Cons.splib
            |       splib_all_ipsc_Cons.sptxt
            |       splib_all_ipsc_Cons.TraML
            |       splib_all_ipsc_Cons.tsv
            |
            +---all_ipsc_Tissues
            |       splib_all_ipsc_Tissues_Cons.pepidx
            |       splib_all_ipsc_Tissues_Cons.spidx
            |       splib_all_ipsc_Tissues_Cons.splib
            |       splib_all_ipsc_Tissues_Cons.sptxt
```

```
|       splib_all_ipsc_Tissues_Cons.TraML
|       splib_all_ipsc_Tissues_Cons.tsv
|
+---day6
|   +---splib
|   |       splib_Day6_Cons.pepidx
|   |       splib_Day6_Cons.spidx
|   |       splib_Day6_Cons.splib
|   |       splib_Day6_Cons.sptxt
|   |       splib_Day6_Cons.TraML
|   |       splib_Day6_Cons.tsv
|   |       splib_Day6_noCons.pepidx
|   |       splib_Day6_noCons.spidx
|   |       splib_Day6_noCons.splib
|   |       splib_Day6_noCons.sptxt
|   |
|   \---Xtandem
|           .gitignore
|           Day_6_DDA_BAND_01.mzid
|           Day_6_DDA_BAND_01.mzXML
|           Day_6_DDA_BAND_01.pep.xml
|           Day_6_DDA_BAND_01.t.xml
|           ...
|           interact.pep-MODELS.html
|           interact.pep.xml
|           interact.pep.xml.index
|           interact_Proph.pep.xml
|           interact_tmp.pep.xml.pIstats
|           mayuCutoff.txt
|           mayuOut_main_1.07.csv
|           mayuOut_main_1.07.txt
|           mayuOut_psm_protFDR0.01_t_1.07.csv
|
+---ipsc
|   +---splib
|   |       splib_iPSC_Cons.pepidx
|   |       splib_iPSC_Cons.spidx
|   |       splib_iPSC_Cons.splib
|   |       splib_iPSC_Cons.sptxt
|   |       splib_iPSC_Cons.TraML
|   |       splib_iPSC_Cons.tsv
|   |       splib_iPSC_noCons.pepidx
|   |       splib_iPSC_noCons.spidx
|   |       splib_iPSC_noCons.splib
|   |       splib_iPSC_noCons.sptxt
|   |
|   \---Xtandem
|           interact.pep-MODELS.html
|           interact.pep.xml
|           interact.pep.xml.index
|           interact_Proph.pep.xml
|           interact_tmp.pep.xml.pIstats
|           iPSC_BAND_1.mzid
```

```
|           iPSC_BAND_1.mzXML
|           iPSC_BAND_1.pep.xml
|           iPSC_BAND_1.t.xml
|           ...
|           mayuCutoff.txt
|           mayuOut_main_1.07.csv
|           mayuOut_main_1.07.txt
|           mayuOut_psm_protFDR0.01_t_1.07.csv
|
+---mef
|   +---splib
|   |       splib_MEFs_Cons.pepidx
|   |       splib_MEFs_Cons.spidx
|   |       splib_MEFs_Cons.splib
|   |       splib_MEFs_Cons.sptxt
|   |       splib_MEFs_Cons.TraML
|   |       splib_MEFs_Cons.tsv
|   |       splib_MEFs_noCons.pepidx
|   |       splib_MEFs_noCons.spidx
|   |       splib_MEFs_noCons.splib
|   |       splib_MEFs_noCons.sptxt
|   |
|   \---Xtandem
|           interact.pep-MODELS.html
|           interact.pep.xml
|           interact.pep.xml.index
|           interact_Proph.pep.xml
|           interact_tmp.pep.xml.pIstats
|           mayuCutoff.txt
|           mayuOut_main_1.07.csv
|           mayuOut_main_1.07.txt
|           mayuOut_psm_protFDR0.01_t_1.07.csv
|           MEFs_BAND_1.mzid
|           MEFs_BAND_1.mzXML
|           MEFs_BAND_1.pep.xml
|           MEFs_BAND_1.t.xml
|           ...
|
+---mixed
|   +---splib
|   |       splib_Mixed_Cons.pepidx
|   |       splib_Mixed_Cons.spidx
|   |       splib_Mixed_Cons.splib
|   |       splib_Mixed_Cons.sptxt
|   |       splib_Mixed_Cons.TraML
|   |       splib_Mixed_Cons.tsv
|   |       splib_Mixed_noCons.pepidx
|   |       splib_Mixed_noCons.spidx
|   |       splib_Mixed_noCons.splib
|   |       splib_Mixed_noCons.sptxt
|   |
|   \---Xtandem
|           BAND_1.mzid
```

```
    |           BAND_1.mzXML
    |           BAND_1.pep.xml
    |           BAND_1.t.xml
    |           ...
    |           interact.pep-MODELS.html
    |           interact.pep.xml
    |           interact.pep.xml.index
    |           interact_Proph.pep.xml
    |           interact_tmp.pep.xml.pIstats
    |           mayuCutoff.txt
    |           mayuOut_main_1.07.csv
    |           mayuOut_main_1.07.txt
    |           mayuOut_psm_protFDR0.01_t_1.07.csv
    |
    \---tissues
        +---splib
        |       splib_tissue_Cons.pepidx
        |       splib_tissue_Cons.spidx
        |       splib_tissue_Cons.splib
        |       splib_tissue_Cons.sptxt
        |       splib_tissue_Cons.TraML
        |       splib_tissue_Cons.tsv
        |       splib_tissue_noCons.pepidx
        |       splib_tissue_noCons.spidx
        |       splib_tissue_noCons.splib
        |       splib_tissue_noCons.sptxt
        |
        \---Xtandem
                ErikM_S1406_034.mzid
                ErikM_S1406_034.mzXML
                ErikM_S1406_034.pep.xml
                ErikM_S1406_034.t.xml
                ...
                interact.pep-MODELS.html
                interact.pep.xml
                interact.pep.xml.index
                interact_Proph.pep.xml
                interact_tmp.pep.xml.pIstats
                mayuCutoff.txt
                mayuOut_main_1.07.csv
                mayuOut_main_1.07.txt
                mayuOut_psm_protFDR0.01_t_1.07.csv
+----ini
+----PXD002896
+----PXD017014
+----iPSCwindowSpecLib.mk
```

Each of the separate libraries are constructed in their own directory `day6` `mef` `ipsc` and `tissues` respectivly. Each of these directries contains a dir `Xtandem` containing all the intermediate files including Xtandem searches, and a `splib` directory containing the spectral library files. The additional directories `all_ipsc` and `all_ipsc_Tissues` contain consensus spectral library files for the combined cell lines, and combined cell line and tissue data respectivly. It is these final directories that are likely to be most useful. The entire `lib` directory occupies about 185 GB of disk space and the complete directory structure occupies about 495 GB.

# Documented Makefile

This section documents each section of the make file used to produce the library. In fact the lines in the makefile are written directly into a `.Rmd` file which is subsequently processed to produce both documentations and makefile. So the lines of the code below are identical to those in `iPSCwindowSpecLib.mk`. This should make it easy to find an edit any required lines in `iPSCwindowSpecLib.mk`.

The program `make` is well documented. Make is principally used to compile software but is also useful to document bioinformatics pipelines. The principle of make is that the program is supplied with a set of rules for converting "prerequisites" into "targets" by means of a "recipy". In this case the targets are a set of spectral libraries and the source files are MS datasets, fasta protein sequence libraries etc. Recipies take the general form:

```
target: prerequisite
  program -in prerequisite -out target
```

Recipies can be written in any order and `make` is able to chain them together to convert source files to target files via intermediate files. The following make file has been written with recipies in the sequence in which they would be run through the pipeline. `Make` will parallelise the processing of multiple files to make full use of a systems compute resource and speed pipeline completion.

Make is able to substitute text for macros which are defined `myMacro=text` or `myMacroList:=element_01 element_02` and then called `${myMacro}` or `${myMacroList}`. It also has a set of built in functions that are called in the general form: `$(function_name variable,param,param...)` which are able to manipulate variables, for example to change a file suffix, or change or remove a directory path. In this way it is possible to write rules to process raw data files into a final output. Each of the `make` functions used for this project are described below with links to the documentation.

## Specify the Shell

Each recipe in the make file is run in its own "shell", that is a separate comand line environment. On windows there is a choice of shell. For simplicity of re-use the windows `cmd.exe` program was chosen which means `dos` comands such as `copy` and `ren` are used which are not present in alternative shells such as `bash`. It is likley the script could be re-written for `bash` and the Windows Subsystem for Linux by changing file path spesification and replacing dos spesific comands, but that has not been attempted here. To ensure the correct shell is used it is set explicity by the `SHELL` variable at the top of the makefile.

```
SHELL=C:/Windows/System32/cmd.exe
```

## Set Shell Environment

Programs in the `Trans Proteomics Pipeline` call other programs in the pipeline and perl scripts when run. In order for these programs to be available they must be on the `PATH` for the shell spesified above. To set the `PATH` the `export` directive is used to set the `PATH` for all shells. This line should be edited to spesify the location of `\TPP\perl\bin` and `\TPP\bin` on the system.

When the Trans Proteomics Pipeline calls `perl` it may return a "locale" error which will also stop `make` processing more results. Expicilty setting the locale to the default "C" locale by exporting `LC_ALL=C` to the shell prevents this error.

```
# Make runs each recipy in a separate shell.
# It is critical that TPP programs are able to search
# the path of this shell for executables. The following "export" statement ensure
# these are available
export PATH=C:\TPP\perl\bin;C:\TPP\bin
# The following export sets the local in the shell to "C" which is
# suitable for processing and prevents spurious error messages
```

```
# that may be returend by perl.
export LC_ALL=C
```

## Set Explicit Paths to Executables

Explicit control of software versions is assured by assigning the full path of each executable to a macro for each of the programs required to produce the libraries. These must be edited to be correct for the system on which the make script is to be run. Setting them explicity should also support reproducability since the version of software used here may be installed along side subsequent releases of the software buing used in future active research. Checking these are correct also acts as a check list to ensure system is set up correctly.

```
# Paths to executables.
# The path on a windows PC may have several versions of these tools installed in
# several locations in various orders on the path. Setting them explicity
# ensures the expected versions are used for processing.
sed_path="C:\Rtools\bin\sed.exe"
awk_path="C:\Rtools\bin\gawk.exe"
DecoyDatabase_path="C:\Program Files\OpenMS-2.4.0\bin\DecoyDatabase.exe"
spectrast_path=\
"C:\Program Files\OpenMS-2.4.0\share\OpenMS\THIRDPARTY\SpectraST\spectrast.exe"
tandem_path="C:\Program Files\tandem-win-17-02-01-4\bin\tandem.exe"
msconvert_path=\
"C:\Program Files\ProteoWizard\ProteoWizard 3.0.20002.36ad78f14\msconvert.exe"
qtofpeakpicker_path=\
"C:\Program Files\ProteoWizard\ProteoWizard 3.0.20002.36ad78f14\qtofpeakpicker.exe"
Tandem2XML_path="C:\TPP\bin\Tandem2XML.exe"
xinteract_path="C:\TPP\bin\xinteract.exe"
PeptideProphet_path="C:\TPP\bin\PeptideProphetParser.exe"
perl_path="C:\TPP\perl\bin\perl.exe"
mayu_path="C:\TPP\bin\Mayu.pl"
idconvert_path="C:\TPP\bin\idconvert.exe"
python_path="C:\ProgramData\Miniconda3\python.exe"
spectrast2tsv_path="C:\ProgramData\Miniconda3\Scripts\spectrast2tsv.py"
TargetedFileConverter_path="C:\Program Files\OpenMS-2.4.0\bin\TargetedFileConverter.exe"

# java and mzidlib are required to generate mzid files primarily for submission to PRIDE.
# If they are not required, coment out these lines and mzid files will not be generated.
# Be sure to give path to 64 bit not 32 bit java as the latter
# can not allocate sufficient memory.
java_path="C:\Program Files\Java\jre1.8.0_231\bin\java.exe"
mzidlib_path="C:\ProteoAnnotator-1.7.86\mzidlib-1.7\mzidlib-1.7.jar"
```

## Raw Data Files

The input files for the spectral library comprise the mass spectrometry data files containing spectral data and the fasta database containing the sequences against which they will be searched.

The raw data obtained from mouse cell cultures undergoing conversion from epithelial cells to pluripotent stem cells aquired on Sciex 6600 triple-TOF systems is stored in pairs of `.wiff` and `.wiff.scan` files. The programs required to convert these data files require the location of only the `.wiff` file so only these are provided. The names of the files from each of the time points are divided into separate groups to enable production of separate libraries. The directory in which these files are stored is stored in `wiffDir` and is used later to locate the files.

Note the extensive use of \ to break a line. This is the accepted way to split lines in a makefile. All elements

on successive lines broken by \ should be considered as on the same logical line. Lines are split here for legibility and to fit the page width of the A4 pdf document. Not the occasional use of `$\` which performs a line break without introducing a space, that is required for several `TPP` programs which do not allow spaces between switches and strings in their comand line invocation.

```
# Wiff file dir
wiffDir = PXD017014

# Find the full list of wiff files in directory
wiffFiles_Day6:=\
Day_6_DDA_BAND_01.wiff Day_6_DDA_BAND_02.wiff Day_6_DDA_BAND_03.wiff \
Day_6_DDA_BAND_04.wiff Day_6_DDA_BAND_05.wiff Day_6_DDA_BAND_06.wiff \
Day_6_DDA_BAND_07.wiff Day_6_DDA_BAND_08.wiff Day_6_DDA_BAND_09.wiff \
Day_6_DDA_BAND_10.wiff Day_6_DDA_BAND_11.wiff Day_6_DDA_BAND_12.wiff \
Day_6_DDA_BAND_13.wiff Day_6_DDA_BAND_14.wiff Day_6_DDA_BAND_15.wiff \
Day_6_DDA_BAND_16.wiff Day_6_DDA_BAND_17.wiff Day_6_DDA_BAND_18.wiff \
Day_6_DDA_BAND_19.wiff Day_6_DDA_BAND_20.wiff Day_6_DDA_BAND_21.wiff \
Day_6_DDA_BAND_22.wiff Day_6_DDA_BAND_23.wiff Day_6_DDA_BAND_24.wiff \
Day_6_DDA_BAND_25.wiff Day_6_DDA_BAND_26.wiff Day_6_DDA_BAND_27.wiff \
Day_6_DDA_BAND_28.wiff Day_6_DDA_BAND_29.wiff Day_6_DDA_BAND_30.wiff

wiffFiles_iPSC:=\
iPSC_BAND_1.wiff iPSC_BAND_10.wiff iPSC_BAND_11.wiff iPSC_BAND_12.wiff \
iPSC_BAND_13.wiff iPSC_BAND_14.wiff iPSC_BAND_15.wiff iPSC_BAND_16.wiff \
iPSC_BAND_17.wiff iPSC_BAND_18.wiff iPSC_BAND_19.wiff iPSC_BAND_2.wiff  \
iPSC_BAND_20.wiff iPSC_BAND_21.wiff iPSC_BAND_22.wiff iPSC_BAND_23.wiff \
iPSC_BAND_24.wiff iPSC_BAND_25.wiff iPSC_BAND_26.wiff iPSC_BAND_27.wiff \
iPSC_BAND_28.wiff iPSC_BAND_29.wiff iPSC_BAND_3.wiff iPSC_BAND_30.wiff \
iPSC_BAND_4.wiff iPSC_BAND_5.wiff iPSC_BAND_6.wiff iPSC_BAND_7.wiff \
iPSC_BAND_8.wiff iPSC_BAND_9.wiff


wiffFiles_MEFs:=\
MEFs_BAND_1.wiff MEFs_BAND_10.wiff MEFs_BAND_11.wiff MEFs_BAND_12.wiff \
MEFs_BAND_13.wiff MEFs_BAND_14.wiff MEFs_BAND_15.wiff MEFs_BAND_16.wiff \
MEFs_BAND_17.wiff MEFs_BAND_18.wiff MEFs_BAND_19.wiff MEFs_BAND_2.wiff \
MEFs_BAND_20.wiff MEFs_BAND_21.wiff MEFs_BAND_22.wiff MEFs_BAND_23.wiff \
MEFs_BAND_24.wiff MEFs_BAND_25.wiff MEFs_BAND_26.wiff MEFs_BAND_27.wiff \
MEFs_BAND_28.wiff MEFs_BAND_29.wiff MEFs_BAND_3.wiff MEFs_BAND_30.wiff \
MEFs_BAND_4.wiff MEFs_BAND_5.wiff MEFs_BAND_6.wiff MEFs_BAND_7.wiff \
MEFs_BAND_8.wiff MEFs_BAND_9.wiff

wiffFiles_Mixed:=\
BAND_1.wiff BAND_10.wiff BAND_11.wiff BAND_12.wiff BAND_13.wiff \
BAND_14.wiff BAND_15.wiff BAND_16.wiff BAND_17.wiff BAND_18.wiff \
BAND_19.wiff BAND_2.wiff BAND_20.wiff BAND_21.wiff BAND_22.wiff \
BAND_23.wiff BAND_24.wiff BAND_25.wiff BAND_26.wiff BAND_27.wiff \
BAND_28.wiff BAND_29.wiff BAND_3.wiff BAND_30.wiff BAND_4.wiff \
BAND_5.wiff BAND_6.wiff BAND_7.wiff BAND_8.wiff BAND_9.wiff
```

Raw data from mouse tissue should be in directory `<baseDir>\PXD002896`. The files are listed here and the directory attached using the function `addprefix`.

```
rawDir=PXD002896
```

```
rawFiles_tissue:=\
ErikM_S1406_034.raw ErikM_S1406_035.raw ErikM_S1406_036.raw \
ErikM_S1406_037.raw ErikM_S1406_038.raw ErikM_S1406_039.raw \
ErikM_S1406_040.raw ErikM_S1406_041.raw ErikM_S1406_042.raw \
ErikM_S1406_043.raw ErikM_S1406_067.raw ErikM_S1406_068.raw \
ErikM_S1406_069.raw ErikM_S1406_070.raw ErikM_S1406_071.raw \
ErikM_S1406_072.raw ErikM_S1406_073.raw ErikM_S1406_074.raw \
ErikM_S1406_075.raw ErikM_S1406_076.raw ErikM_S1406_152.raw \
ErikM_S1406_153.raw ErikM_S1406_154.raw ErikM_S1406_155.raw \
ErikM_S1406_156.raw ErikM_S1406_157.raw ErikM_S1406_158.raw \
ErikM_S1406_159.raw ErikM_S1406_160.raw ErikM_S1406_161.raw \
ErikM_S1406_163.raw ErikM_S1406_164.raw ErikM_S1406_165.raw \
ErikM_S1406_166.raw ErikM_S1406_167.raw ErikM_S1406_168.raw \
ErikM_S1406_169.raw ErikM_S1406_170.raw ErikM_S1406_171.raw \
ErikM_S1406_172.raw Erik_S1407_013.raw Erik_S1407_014.raw   \
Erik_S1407_015.raw Erik_S1407_016.raw Erik_S1407_017.raw \
Erik_S1407_018.raw Erik_S1407_019.raw Erik_S1407_020.raw \
Erik_S1407_021.raw Erik_S1407_022.raw Erik_S1407_024.raw \
Erik_S1407_025.raw Erik_S1407_026.raw Erik_S1407_027.raw \
Erik_S1407_028.raw Erik_S1407_029.raw Erik_S1407_030.raw \
Erik_S1407_031.raw Erik_S1407_032.raw Erik_S1407_033.raw \
Erik_S1407_035.raw Erik_S1407_036.raw Erik_S1407_037.raw \
Erik_S1407_038.raw Erik_S1407_039.raw Erik_S1407_040.raw \
Erik_S1407_041.raw Erik_S1407_042.raw Erik_S1407_043.raw \
Erik_S1407_044.raw Erik_S1407_046.raw Erik_S1407_047.raw \
Erik_S1407_048.raw Erik_S1407_049.raw Erik_S1407_050.raw \
Erik_S1407_051.raw Erik_S1407_052.raw Erik_S1407_053.raw \
Erik_S1407_054.raw Erik_S1407_055.raw Erik_S1407_057.raw \
Erik_S1407_058.raw Erik_S1407_059.raw Erik_S1407_060.raw \
Erik_S1407_061.raw Erik_S1407_062.raw Erik_S1407_063.raw \
Erik_S1407_064.raw Erik_S1407_065.raw Erik_S1407_066.raw \
Erik_S1407_068.raw Erik_S1407_069.raw Erik_S1407_070.raw \
Erik_S1407_071.raw Erik_S1407_072.raw Erik_S1407_073.raw \
Erik_S1407_074.raw Erik_S1407_075.raw Erik_S1407_076.raw \
Erik_S1407_077.raw Erik_S1407_079.raw Erik_S1407_080.raw \
Erik_S1407_081.raw Erik_S1407_082.raw Erik_S1407_083.raw \
Erik_S1407_084.raw Erik_S1407_085.raw Erik_S1407_086.raw \
Erik_S1407_087.raw Erik_S1407_088.raw Erik_S1407_090.raw \
Erik_S1407_091.raw Erik_S1407_092.raw Erik_S1407_093.raw \
Erik_S1407_094.raw Erik_S1407_095.raw Erik_S1407_096.raw \
Erik_S1407_097.raw Erik_S1407_098.raw Erik_S1407_099.raw \
Erik_S1408_076.raw Erik_S1408_077.raw Erik_S1408_079.raw \
Erik_S1408_080.raw Erik_S1408_081.raw Erik_S1408_082.raw \
Erik_S1408_083.raw Erik_S1408_084.raw Erik_S1408_085.raw \
Erik_S1408_086.raw Simon_S1411_271.raw Simon_S1411_272.raw \
Simon_S1411_273.raw Simon_S1411_274.raw Simon_S1411_275.raw \
Simon_S1411_277.raw Simon_S1411_278.raw Simon_S1411_279.raw \
Simon_S1411_280.raw Simon_S1411_281.raw Simon_S1411_283.raw \
Simon_S1411_284.raw Simon_S1411_285.raw Simon_S1411_286.raw \
Simon_S1411_287.raw Simon_S1411_288.raw Simon_S1411_290.raw \
Simon_S1411_291.raw Simon_S1411_292.raw Simon_S1411_293.raw \
Simon_S1411_294.raw Simon_S1411_295.raw Simon_S1411_297.raw \
Simon_S1411_298.raw Simon_S1411_299.raw Simon_S1411_300.raw
```

```
rawFiles_tissue_dir:=$(addprefix .\${rawDir}\, ${rawFiles_tissue})
```

### Protein Sequence Database (.fasta) Files

The library search requires a fasta library complete with the mouse sequences, the iRT sequences and a decoy library. The location of mouse and iRT database are set here. The name of the fasta file actually used in the search is set as `fastaForTandem` using the `subst` function to replace the `.fasta` suffix with `_rev.fasta`. This library will be built from the input fasta files and then subsequently used for the search. The marker for decoy database is also set here as `reverse_` this is used during construction of database for search and subsequently to filter decoy proteins from the search results.

There are four fasta databases used for this search: the mouse proteome; the iRT sequences and two common contaminants databases. These databases are combined into two databases: `fastaForTandem` for searching which includes iRT, contaminants and a decoy database; and `fastaForLabel`, a pure mouse and iRT database excluding decoy and contraminants for re-annotating the final spectral library. The libraries are combined in subsequent comands.

The strings used to denote decoy and contaminant entries in the `fasta` databases are also defined. Finnaly in this section the file containing the iRT indexes is set.

```
# Protein fasta files

fastaBase = .\ini\uniprot-proteome3AUP000000589.fasta
fastaSwissProt = .\ini\uniprot-proteomeUP000000589_swissProt.fasta
fasta_iRT = .\ini\irtfusion.fasta
fasta_cRAP = .\ini\crap.fasta
fasta_MPI = .\ini\contaminants.fasta
fastaForTandem = $(subst .fasta,_rev.fasta,${fastaBase})
fastaForLabel = $(subst .fasta,_iRT.fasta,${fastaBase})

decoy_string = reverse_
cont_string = cont_

# Either biognosis iRT of Sciex pepCal retention time index peptides may be used.
RTindex = .\ini\iRTPeptides.txt
```

### Lists for Target and Intermediate Files

The targets for the makefile include the `.splib/.sptxt` files produced by spectrast and the `.tsv` and `.TraML` derived from them. Optinal additional targets include the `.mzid` files required for pride submission. The path to producing these files requires production of intermediate files `.mzXML` and various `.pep.xml` files. All these files are listed here. Eash list is used to spesify targets below.

```

# Spesify mzXML files including target directory by swapping .wiff or .raw suffix for
# .mzXML and adding dir prefix.
mzxml_CentFiles_Day6  :=$(addprefix .\lib\day6\Xtandem\,$(wiffFiles_Day6:.wiff=.mzXML))
mzxml_CentFiles_iPSC  :=$(addprefix .\lib\ipsc\Xtandem\,$(wiffFiles_iPSC:.wiff=.mzXML))
mzxml_CentFiles_MEFs  :=$(addprefix .\lib\mef\Xtandem\,$(wiffFiles_MEFs:.wiff=.mzXML))
mzxml_CentFiles_Mixed :=$(addprefix .\lib\mixed\Xtandem\,$(wiffFiles_Mixed:.wiff=.mzXML))
mzxml_CentFiles_tissue :=$(addprefix .\lib\tissues\Xtandem\,$(rawFiles_tissue:.raw=.mzXML))

# Concatenate lists of centroided files together.
all_CentFiles:= ${mzxml_CentFiles_Day6} ${mzxml_CentFiles_iPSC} \
```

```
            ${mzxml_CentFiles_MEFs} ${mzxml_CentFiles_Mixed} \
            ${mzxml_CentFiles_tissue}


# spesify the names of groups of files to enable xinteract to operate
# on each group separatly (see below).
list_groups:= day6 ipsc mef mixed tissues

# Determin names of pep.xml files
# ifdef comands ensure this is only done if java and mzidlib are spesified above.
# if not this section will not be defined and recipies to produce mzid files not run.
pep_xml_files:= $(all_CentFiles:.mzXML=.pep.xml)
ifdef java_path
ifdef mzidlib_path
mzid_files:= $(all_CentFiles:.mzXML=.mzid)
endif
endif

# Spesify xtandem search combined interact.pep.xml files.
xtand_Day6  = .\lib\day6\Xtandem\interact.pep.xml
xtand_iPSC  = .\lib\ipsc\Xtandem\interact.pep.xml
xtand_MEFs  = .\lib\mef\Xtandem\interact.pep.xml
xtand_Mixed = .\lib\mixed\Xtandem\interact.pep.xml
xtand_tissue = .\lib\tissues\Xtandem\interact.pep.xml

xtand_all:= ${xtand_Day6} ${xtand_iPSC} ${xtand_MEFs} \
                        ${xtand_Mixed} ${xtand_tissue}

# Spesify names of files prcessed by peptide prophet.
xtand_Day6_Proph  = .\lib\day6\Xtandem\interact_Proph.pep.xml
xtand_iPSC_Proph  = .\lib\ipsc\Xtandem\interact_Proph.pep.xml
xtand_MEFs_Proph  = .\lib\mef\Xtandem\interact_Proph.pep.xml
xtand_Mixed_Proph = .\lib\mixed\Xtandem\interact_Proph.pep.xml
xtand_tissue_Proph = .\lib\tissues\Xtandem\interact_Proph.pep.xml

xtand_all_Proph:= ${xtand_Day6_Proph} ${xtand_iPSC_Proph} ${xtand_MEFs_Proph} \
                                    ${xtand_Mixed_Proph} ${xtand_tissue_Proph}

# Spesify no consensus splib files
splib_Day6_noCons  = .\lib\day6\splib\splib_Day6_noCons.splib
splib_iPSC_noCons  = .\lib\ipsc\splib\splib_iPSC_noCons.splib
splib_MEFs_noCons  = .\lib\mef\splib\splib_MEFs_noCons.splib
splib_Mixed_noCons = .\lib\mixed\splib\splib_Mixed_noCons.splib
splib_tissue_noCons = .\lib\tissues\splib\splib_tissue_noCons.splib

allSplibNoCons := ${splib_Day6_noCons} ${splib_iPSC_noCons} ${splib_MEFs_noCons} \
                                    ${splib_Mixed_noCons} ${splib_tissue_noCons}

#Spesify splib consensus files.
splib_Day6  = .\lib\day6\splib\splib_Day6_Cons.splib
splib_iPSC  = .\lib\ipsc\splib\splib_iPSC_Cons.splib
splib_MEFs  = .\lib\mef\splib\splib_MEFs_Cons.splib
splib_Mixed = .\lib\mixed\splib\splib_Mixed_Cons.splib
```

```
splib_tissue = .\lib\tissues\splib\splib_tissue_Cons.splib
splib_all_ipsc = .\lib\all_ipsc\splib_all_ipsc_Cons.splib
splib_all_ipsc_Tissues = .\lib\all_ipsc_Tissues\splib_all_ipsc_Tissues_Cons.splib
splib_all_ipsc_Tissues_SP = .\lib\all_ipsc_Tissues\splib_all_ipsc_Tissues_Cons_SP.splib

all_iPSC_Splib := ${splib_Day6} ${splib_iPSC} ${splib_MEFs} ${splib_Mixed}
allSplib := ${all_iPSC_Splib} ${splib_tissue}
allSplibIncCombined := ${allSplib} ${splib_all_ipsc_Tissues} ${splib_all_ipsc} ${splib_all_ipsc_Tissues_

# Spesify .tsv files for each .splib file
allFiltered := $(allSplibIncCombined:.splib=.tsv)
allFiltered_peakview := $(allSplibIncCombined:.splib=_peakview.tsv)

# Spesify .TraML file for each .splib file
allTraML:= $(allSplibIncCombined:.splib=.TraML)
```

## Make All Targets

The following section sets the special targets for make. The `.PHONY` target indicates targets that are not real files. It is common to set `all` as a phony target and then list all real targets under all, as here the `.TraML` and `.tsv` files defined above in `${allTraML}` and `${allFiltered}` are entered under all. The `.mzid` files are also included in `all` as they are side products note required to make the `.TraML` and `.tsv` files themselves. To prevent production of `.mzid` files remove `${mzid_files}`.

```
.PHONY: all


all: ${allTraML} ${allFiltered} ${allFiltered_peakview} ${mzid_files} ${allSplibIncCombined}
```

## Convert Raw Mass Spectrometry Data to mzXML

Each of the required libraries must now be build from the `.wiff`, `.wiff.scan` and `.raw` files. Intermediate files must be created in the correct location in the directory structure produced above. However each rule for producing each required intermediate file should be entered only once, both to ensure a single set of parameters are used consistantly across the process and to facilitate comprehention and reuse of the code. For this the gmake `define` function is used to describe canned recipes which are then made available as a separate recipy for each file using the `call` function.

All the mass spectrometry data files in their native formats must be converted to `.mzXML` format prior to searching The Sciex paired `.wiff`, `.wiff.scan` are converted in a two stage process: Firstly the `qtofpeakpicker.exe` program from Proteowizard creates an unfiltered and so very large `.mzxml` file. Then msconvert from proteowizard creates a smaller file filtered for the top 150 peaks per spectrum.

This is the first example in this makefile of a "canned" recipe. The recipe `wiff2mzxml_Rule` is first defined with numbered macros for substitution `${1}` and `${2}`. The `foreach` function then creates a version of the rule for each entry `wiffFile` in a list, for example `${wiffFiles_Day6}`. The rules are constructed by `call` which takes the predefined rule `wiff2mzxml_Rule` and replaces `${1}` and `${2}` with the target `.mzXML` and the prerequisit `.wiff` file repectivly. The `eval` function then turns the rule contructed by `call` into a real makefile construct. For debugging, or for interest, replace the `eval` with the `info` function to output all the recipies to the console. Note replacing `eval` with `info` will prevent libraries being built as rules will not be available.

This structure is used repeatedly through the rest of the makefile and will not be described in such detail again.

```
define wiff2mzxml_Rule
${1} : ${2}
    if not exist $(dir ${1}) mkdir $(dir ${1})
    ${qtofpeakpicker_path} --resolution=25000 \
                            --area=1 --threshold=1 \
                            --smoothwidth=1.1 \
                            --in=${2} \
                            --out=$(basename ${1})_large.mzxml
    ${msconvert_path} $(basename ${1})_large.mzxml --mzXML \
                    --filter "threshold count 150 most-intense" \
                    -o $(dir ${1}) \
                    --outfile $(notdir ${1})
    del /F $(basename ${1})_large.mzxml
endef


# Day six rules:
$(foreach wiffFile, ${wiffFiles_Day6},\
    $(eval \
        $(call wiff2mzxml_Rule,\
            .\lib\day6\Xtandem\$(wiffFile:.wiff=.mzXML),${wiffDir}\$(wiffFile)\
            )\
        )\
)


# ipsc rules:
$(foreach wiffFile, ${wiffFiles_iPSC},\
    $(eval \
        $(call wiff2mzxml_Rule,\
            .\lib\ipsc\Xtandem\$(wiffFile:.wiff=.mzXML),${wiffDir}\$(wiffFile)\
            )\
        )\
)

# mef rules:
$(foreach wiffFile, ${wiffFiles_MEFs},\
    $(eval \
        $(call wiff2mzxml_Rule,\
            .\lib\mef\Xtandem\$(wiffFile:.wiff=.mzXML),${wiffDir}\$(wiffFile)\
        )\
    )\
)

# mixed rules:
$(foreach wiffFile, ${wiffFiles_Mixed},\
    $(eval \
        $(call wiff2mzxml_Rule,\
            .\lib\mixed\Xtandem\$(wiffFile:.wiff=.mzXML),${wiffDir}\$(wiffFile)\
        )\
    )\
)
```

The thermo `.raw` files are converted in a single step using msconvert from proteowizard. Note that the switch

`--singleThreaded` was required to prevent errors on our system.

```
define raw2mzxml_Rule
${1} : ${2}
    if not exist $(dir ${1}) mkdir $(dir ${1})
    ${msconvert_path} ${2} --singleThreaded --mzXML  --filter "peakPicking true 1-" \
    --filter "threshold count 150 most-intense" \
    -o $(dir ${1}) \
    --outfile $(notdir ${1})
endef

# tissue rules
$(foreach rawFile, ${rawFiles_tissue_dir},\
    $(eval \
        $(call raw2mzxml_Rule,\
            $(addprefix .\lib\tissues\Xtandem\,\
            $(notdir $(rawFile:.raw=.mzXML))),${rawFile})\
    )\
)
```

## Combine Protein Sequence Databases and Add Decoys

As set up the `ini` directory contains the uniprot mouse database, two databases of common contaminants and one containing iRT peptides. These are concatenated into a single fasta file as follows. First a "clean" mouse datbase is created by removing the protein description from each entry header, but leaving it's accession number. This is required to prevent a clash with the pride parser and may be removes if data is not to be uploaded to pride. The marker `cont_` is pre-pended to each header line in the contaminants databases. The fasta databases are concatenated and a reverse decoy database is appended for the search. An additional fasta database containing just the clean mouse and the iRT databases, the spectral libraries will be re-annotated against this database to ensure no decoy or contaminant spectra progress to final output.

```
$(fastaBase:.fasta=_clean.fasta) : ${fastaBase}
    ${sed_path} -e 's/[[:blank:]].*//g' $< > $@


$(fasta_cRAP:.fasta=_clean.fasta) : ${fasta_cRAP}
    ${sed_path} -e 's/^>/^>${cont_string}/g' $< > $@


$(fasta_MPI:.fasta=_clean.fasta) : ${fasta_MPI}
    ${sed_path} -e 's/^>/^>${cont_string}/g' $< > $@


${fastaForTandem} : $(fastaBase:.fasta=_clean.fasta) \
                    $(fasta_cRAP:.fasta=_clean.fasta) \
                    $(fasta_MPI:.fasta=_clean.fasta) ${fasta_iRT}
    ${DecoyDatabase_path} -in $^ -out $@ -decoy_string ${decoy_string}


${fastaForLabel} : $(fastaBase:.fasta=_clean.fasta) ${fasta_iRT}
    type $(fastaBase:.fasta=_clean.fasta) ${fasta_iRT} > ${fastaForLabel}
```

## X!Tandem Search

Now that all the required spectra and fasta files are available the xtandem search can be run. The xtandem program requires a parameter file in `.xml` format that spesifies settings for the analysis and the location of input spectrum and output `.t.xml` result file. The `xtandem` program is well documented here and here. Essentially the program takes a single input `.xml` file which spesifies a spectrum file, sequence database and search parameters. Here a base `Xtandem_params.xml` file is copied from the `ini` directory to the xtandem processing directory and re-named for the input file. The strings "spectrumFile" and "outFile" in the paramater file are then replaced with the appropriate file locations for the spesific target using `sed`. The `xtandem` search is then run producing a `.t.xml` output file. On windows `sed` produces a backup of the modified file with name SED which rapidly fill the base directory. Any examples of this file type are cleared from the base directory by comand `if exist sed* del sed*` after each search.

```
define Xtandem_Rule
${1} : ${2} .\ini\taxonomy.xml .\ini\default_input.xml .\ini\Xtandem_params.xml ${fastaForTandem}
    if not exist $(dir ${1}) mkdir $(dir ${1})
    copy .\ini\Xtandem_params.xml $(1:.t.xml=_params.xml)
    ${sed_path} -i "s|spectrumFile|$(subst \,\\,${2})|"  $(1:.t.xml=_params.xml)
    ${sed_path} -i "s|outFile|$(subst \,\\,${1})|" $(1:.t.xml=_params.xml)
    ${tandem_path} $(1:.t.xml=_params.xml)
    del /F $(1:.t.xml=_params.xml)
    if exist sed* del sed*
endef

$(foreach centFile, ${all_CentFiles},\
        $(eval \
            $(call Xtandem_Rule,$\
                $(centFile:.mzXML=.t.xml),${centFile}$\
            )\
        )\
)
```

## Converting `.t.xml` Fileto `.mzid` For Upload to Pride

The `.t.xml` result files from xtandem may be converted to mzid files suitable for upload to pride. The following recipy uses `mzid-lib` to produce the mzid files. The `mzid` files are annotated with the correct database file format and mass spec file format ontology. As noted above this is only done if the `${java_path}` and `${mzidlib_path}` are defined.

```
# The following rule will build mzid files
# ontology fasta : MS:1001348
# https://www.ebi.ac.uk/ols/ontologies/MS/terms?obo_id=MS:1001348
# ontology mzxml: MS:1000566
# https://www.ebi.ac.uk/ols/ontologies/MS/terms?obo_id=MS:1000566

define mzid_Rule
${1} : ${2}
    ${java_path} -Xms1500m -Xmx3G -jar ${mzidlib_path} Tandem2mzid ${2} ${1} \
    -outputFragmentation true -decoyRegex ${decoy_string} \
    -databaseFileFormatID MS:1001348  -massSpecFileFormatID MS:1000566 \
    -idsStartAtZero false -compress false
endef

$(foreach mzid, ${mzid_files},\
```

```
        $(eval \
            $(call mzid_Rule,\
                    ${mzid},\
                    $(mzid:.mzid=.t.xml)\
                )\
        )\
)
```

### Combine Search Results and Process Through Trans-Proteomics Pipeline

The downstreem processing pathway requires `.t.xml` xtandem output files to be converted to `.pep.xml` files for the trans-proteomic piepline. This is simply accomplished by running Tandem2XML.exe on each `.t.xml` file.

```
define Xtandem_pep_xml_Rule
${1}: ${2}
    ${Tandem2XML_path} ${2} ${1}
endef

$(foreach centFile, ${all_CentFiles},\
    $(eval \
        $(call Xtandem_pep_xml_Rule,\
                $(centFile:.mzXML=.pep.xml),\
                $(centFile:.mzXML=.t.xml)\
            )\
    )\
)
```

Now there is a `.pep.xml` file available for each fraction the fractions need to be combined into a single file for each time point in conversion from fibroblast to iPSC cells, and for the mouse tissue downloaded from pride. This is achieved by running `xinteract` on sets of .pep.xml search results.

The switches chosen for xinteract are as follows:

**-eT** specify sample enzyme = Trypsin
**-MONO** calculate monoisotopic peptide masses during conversion to pepXML
**-E<experiment_label>** used to commonly label all spectra belonging to one experiment (required by iProphet). In this case the sample that gave rise to the fractions.
**-l<num>** minimum peptide length considered in the analysis (explicity sets the default 7)
**-nP** do not run PeptideProphet (it is run in a separate step subsequently)
**-D<database_path>** specify path to database
**-d<tag>** use decoy hits to pin down the negative distribution. The decoy protein names must begin with (whitespace is not allowed)
**-N<output.pep.xml>** write output to file.

The `foreach` function is made to work slightly differntly here. The function works through every `group` in `${list_groups}` which was defined above. The selected `${group}` variable is then used to spesify the directory the resulting `interact.pep.xml` will drop into and to `filter` the complete list of `${pep_xml_files}` for those in the `${group}` directory. In this way a separate `interact.pep.xml` file is created for each set of search results files.

```
define xinteract_Rule
${1} : ${2}
    if not exist $(dir ${1}) mkdir $(dir ${1})
    ${xinteract_path} -eT \
```

```
                            -MONO \
                            -E${3} \
                            -l7 \
                            -nP \
                            -D${fastaForTandem} \
                            -d${decoy_string} \
                            -N${1} ${2}
endef

$(foreach group, ${list_groups},\
        $(eval \
            $(call xinteract_Rule,$\
                    .\lib\${group}\Xtandem\interact.pep.xml,$\
                    $(filter .\lib\${group}\Xtandem%,${pep_xml_files}),$\
                    ${group}\
                )\
            )\
)
```

After combining and refining the spectra with **xinteract** the peptide-spectra matches are re-scored using peptideprophet. This tool modifies the output of xinteract in place. So to keep a record of intermediate files and so that make's understanding of targets and prerequisits is operable the process is run as follows: 1. Copy output from xinteract to a temperary file with suffix `_tmp.pep.xml`. 2. Run peptide prophet parser on the temporary file. 3. Rename the new file to the target `_Proph.pep.xml` file.

Switches were chosen as follows:

**ACCMASS** Use Accurate Mass model binning (default NO)
**PPM** Use ppm mass error instead of Daltons for mass modeling (default Daltons)
**PI** Enable peptide pI model (default NO)
**MINPROB=<number>** Report results with minimum probability (explicity sets default 0.05)
**DECOY=<decoy_prot_prefix>** semi-supervised mode, protein name prefix to identify Decoy entries set as `${decoy_string}`
**DECOYPROBS** compute possible non-zero probabilities for Decoy entries on the last iteration. Required by Mayu.
**NONPARAM** use semi-parametric modeling, must be used in conjunction with DECOY= option (default NO)
**NONTT** disable NTT enzymatic termini model (default NO)
**IGNORECHG=<charge>** can be used multiple times to specify all charge states to exclude from modeling. Here all charge states but 2 and 3 are excluded.

```
define peptideProphet_Rule
${1} : ${2}
    if not exist $(dir ${1}) mkdir $(dir ${1})
    copy ${2} $(2:.pep.xml=_tmp.pep.xml)
    ${PeptideProphet_path} $(2:.pep.xml=_tmp.pep.xml) \
    ACCMASS PPM PI MINPROB=0.05 DECOY=${decoy_string} \
    DECOYPROBS NONPARAM NONTT IGNORECHG=1 IGNORECHG=4 \
    IGNORECHG=5 IGNORECHG=6 IGNORECHG=7
    ren $(2:.pep.xml=_tmp.pep.xml) $(notdir ${1})
endef

$(foreach prophOut, ${xtand_all_Proph},\
        $(eval \
            $(call peptideProphet_Rule,$\
                    ${prophOut},$\
```

```
                    $(prophOut:_Proph.pep.xml=.pep.xml)$\
                )\
            )\
)
```

## Controlling False Positive Rate

PeptideProphet has controlled the false positive rate at 5% on peptide-spectrum matches. However for a quality spectral library the false discovery rate for proteins must be controlled. The `Mayu` program is run to find the score cutoff required to hold the protein false discovery rate at 1%.

Switches are set as follows:

**-A** The pepXML file
**-M** The output file name base (The element "__psm__protFDR0.01__t__1.07.csv" is added by mayu and so has to be removed from target here.)
**-C** The target decoy search database (as passed to xtandem).
**-E** decoy id prefix (as defined above).
**-G** The maximum mFDR (spectra-peptide match FDR)
**-H** number of mFDR steps (between 0 and that spesified by `-G`)
**-I** Number of missed cleavages used in database search
**-P** select a filtered list of PSM to be printed out.
We have selected protFDR=0.01:t which sets protein false discovery to 1% and prints out target spectral matches. If the FDR were to be changed here the mayu output file name would require corresponding alteration to match.

```

define mayu_Rule
${1} : ${2}
    if not exist $(dir ${1}) mkdir $(dir ${1})
    ${perl_path} ${mayu_path} \
    -A ${2} \
    -M $(subst _psm_protFDR0.01_t_1.07.csv,,${1}) \
    -C ${fastaForTandem} \
    -E ${decoy_string} \
    -G 0.01 \
    -H 49 \
    -I 1 \
    -P protFDR=0.01:t
endef

$(foreach prophOut, ${xtand_all_Proph},\
        $(eval \
            $(call mayu_Rule,\
                $(dir ${prophOut})mayuOut_psm_protFDR0.01_t_1.07.csv,\
                ${prophOut}\
            )\
        )\
)
```

## Building Individual Libraries with Spectrast

Spectral libraries are built using `spectrast`. This is done in two steps. First a library of all spectra is created and the retention times normalised to the iRT. Second a 'consensus' library is created which retains a single consensus spectra for each peptide in the library. In this second stage peptide mappings are refreshed against

the **${fastaForLabel}** database create above and none matching petides discarded. The **${fastaForLabel}** database contains only the mouse database and iRT proteins with no contaminants or decoys, this step results in a clean spectral library.

Within the recipe, prior to building the library, the peptide-spectra cut off required to control the protein FDR has to be colected from the **mayu** output file, which contains all spectra-peptide matches given a protein FDR of 1%. Included in that file is a column of peptide-spectra-match probabilities. The minimum probability score in this column is the minimum score required to give the required protein FDR. The minimum probability is recovered from the file using the **eval** function again, but this time within the recipe so that the comand is only run when the recipe is called. When run the **eval** function sets the **mayuCutoff** variable using **gawk** to run down the fifth column of the mayu output file comparing each encountered probability against the one currently stored as variable **min**, if lower than the current value, the value is updated. Once **gawk** has processed the entire file the last value of **min** is returned as variable mayuCutoff.

Spectrast is then run with the following switches:

**-cN<file>** Spesifies the output file name
**-cICID-QTOF** tag all library spectra as Q-TOF (high-resolution) CID spectra.
**-c_IRR** Regress the real RTs of landmark peptides (i.e. assume they form a straight line).
**-c_IRT<file>** Use landmark peptides in to normalize retention times to iRTs.
**-cP** Include all spectra identified with probability no less than in the library. (from mayu see above)
**-c_BRK** Bracket import: for each confident ID, also search neighboring scans for repeated scans to import.
**-c_BRM** Merge bracketed spectra: merge repeated scans of a bracket into one consensus spectrum for import.
**-c_RDY<prefix>** Remove spectra of decoys, for which proteins have names starting with . Also remove decoy proteins from Protein field for peptides mapped to both target and decoy sequences.

```
define spectrast_Rule
${1} $(splibNoCons:.splib=.sptxt) : ${2} ${3} ${4} $${pep_xml_files}
    if not exist $(dir ${1}) mkdir $(dir ${1})
    $$(eval mayuCutoff := \
    $$(shell ${awk_path} -F, "BEGIN{min=1}{if($$$$5<=min)  min=$$$$5}END{print min}" ${4})\
        )
    $$(file > $$(dir ${4})mayuCutoff.txt,$${mayuCutoff})
    ${spectrast_path} -V \
                -cN$(basename $1) \
                -cICID-QTOF \
                -c_IRR \
                -c_IRT${3} \
                -cP$${mayuCutoff} \
                -c_BRK \
                -c_BRM \
                -c_RDY"${decoy_string}" \
                ${2}
endef

$(foreach splibNoCons, ${allSplibNoCons},\
    $(eval\
        $(call spectrast_Rule,$\
            $(splibNoCons),$\
            $(subst splib,Xtandem,$(dir ${splibNoCons}))interact_Proph.pep.xml,$\
            ${RTindex},$\
            $(subst splib,Xtandem,$(dir ${splibNoCons})mayuOut_psm_protFDR0.01_t_1.07.csv)$\
        )\
    )\
)
```

The spectral library now exists but contains redundent spectra, decoys and contaminants. Spectrast is therefore run a second time to produce consensus spectra and reannotate those spectra against the `.fasta` database comprising just the mouse proteome and the iRT peptides excluding decoy and contaminant peptide. The result is a clean spectral library.

Spectrast is then run with the following switches:

**-cN<file>** Spesifies the output file name
**-cD<file>** Refresh protein mappings against the database in FASTA format.
**-cu** Delete entries whose peptide sequences do not map to any protein during refreshing with -cD option.
**-cAC** Create consensus[1] spectra of all replicate spectra of each peptide ion.

```
define spectrastCons_Rule
${1} $(1:.splib=.sptxt) : ${2} ${fastaForLabel}
    if not exist $(dir ${1}) mkdir $(dir ${1})
    ${spectrast_path} -V \
                -cN$(basename ${1}) \
                -cD${fastaForLabel} \
                -cu \
                -cAC \
                ${2}
endef

$(foreach splibCons,${allSplib},$\
        $(eval\
            $(call spectrastCons_Rule,$\
                ${splibCons},$\
                $(splibCons:_Cons.splib=_noCons.splib)$\
            )\
        )\
)
```

## Combine Libraries

Each individual library is now complete. These are now combined using spectrast to give two consensus libraries. One combines all the cell derived libraries into a single file `splib_all_ipsc_Cons`. The other combines all libraries including both the cell and tissue libraries into a single library `splib_all_ipsc_Tissues_Cons`.

```

.\lib\all_ipsc\splib_all_ipsc_Cons.sptxt \
.\lib\all_ipsc\splib_all_ipsc_Cons.splib : ${all_iPSC_Splib}
    if not exist .\lib\all_ipsc mkdir .\lib\all_ipsc
    ${spectrast_path} -V \
                -cN$(basename $@) \
                -cAC \
                -cJU \
                $(filter %.splib,$^)

#################
#################
#################

.\lib\all_ipsc_Tissues\splib_all_ipsc_Tissues_Cons.sptxt \
.\lib\all_ipsc_Tissues\splib_all_ipsc_Tissues_Cons.splib: ${allSplib}
    if not exist .\lib\all_ipsc_Tissues\ mkdir .\lib\all_ipsc_Tissues
```

```
    ${spectrast_path} -V \
                -cN$(basename $@) \
                -cAC \
                -cJU \
                -cu \
                -cD${fastaForLabel} \
                $(filter %.splib,$^)

#################
#################
#################
```

## Remap Combined Library to SwissProt Libary

Each library is now mapped to the "swissprot" part of the uniprot database. This is a curated sequence database and mapping to it removes redundancy from the spectral library. About 5% of the peptide spectra a removed by this step.

```


$(splib_all_ipsc_Tissues_SP:.splib=.sptxt) ${splib_all_ipsc_Tissues_SP}: \
.\lib\all_ipsc_Tissues\splib_all_ipsc_Tissues_Cons.splib ${fastaSwissProt}
    if not exist .\lib\all_ipsc_Tissues\ mkdir .\lib\all_ipsc_Tissues
    ${spectrast_path} -V \
                -cN$(basename $@) \
                -cAC \
                -cJU \
                -cu \
                -cD${fastaSwissProt} \
                $(filter %.splib,$^)

#################
#################
#################
```

## Conversion of Library into Other Formats

The `.sptxt` spectral library format may be used directly with Skyline. Other formats are required by other software. There are several `.tsv` tab separated value formats available for spectral libraries. In addition to use with spesific processing software these are ameanable to analysis with other tools such as `R`. The python script `spectrast2tsv` is used here to convert the `.sptxt` spectral library to .

`spectrast2tsv` is run with the following switches:

**-l** mass limits
**-s** List of ion series to be used (b and y in this case)
**-x** Fragment ion charge states allowed. (1 and 2)
**-g** List of allowed fragment mass modifications. (loss of water and ammonia)
**-o** Min number of reported ions per peptide/z (explisity set default of 3)
**-n** Max number of reported ions per peptide/z. Default: 20 (explisitly set default 20)
**-p** Maximum error allowed at the annotation of a fragment ion. (explisitly set default 0.05)
**-d** Remove duplicate masses from labeling
**-e** Use theoretical mass
**-k** Select the output provided. In the first case set to `openswath` in one case to enable subsequent `.traML` production. In the second case `peakview` to produce a spectral library for Sciex's Peakview software.

**-w** File containing the swath ranges. This is used to remove transitions with Q3 falling in the swath mass range. (This may need to be adjusted if the library is rebuilt for an alternative SWATH window schema or a non-SWATH usage).

**-a** Output file name

```
define spectrast2tsv_Rule
${1} : ${2} ${3}
    if not exist $(dir ${1}) mkdir $(dir ${1})
    ${python_path} ${spectrast2tsv_path}  \
                        -l 100,2000 \
                        -s b,y \
                        -x 1,2 \
                        -g -17.03,-18.0 \
                        -o 3 \
                        -n 20 \
                        -p 0.05 \
                        -d -e \
                        -k openswath \
                        -w ${3} \
                        -a ${1} \
                        ${2}
endef

$(foreach splib2conv, ${allSplibIncCombined},\
        $(eval \
                $(call spectrast2tsv_Rule,\
                        $(basename ${splib2conv}).tsv,\
                        $(basename ${splib2conv}).sptxt,\
                        .\ini\SWATH_windows.txt\
                    )\
            )\
)
```

```
define spectrast2tsv_peakview_Rule
${1} : ${2} ${3}
    if not exist $(dir ${1}) mkdir $(dir ${1})
    ${python_path} ${spectrast2tsv_path}  \
                        -l 100,2000 \
                        -s b,y \
                        -x 1,2 \
                        -g -17.03,-18.0 \
                        -o 3 \
                        -n 20 \
                        -p 0.05 \
                        -d -e \
                        -k peakview \
                        -w ${3} \
                        -a ${1} \
                        ${2}
endef
```

```
$(foreach splib2conv, ${allSplibIncCombined},\
        $(eval \
                $(call spectrast2tsv_peakview_Rule,\
                        $(basename ${splib2conv})_peakview.tsv,\
                        $(basename ${splib2conv}).sptxt,\
                        .\ini\SWATH_windows.txt \
                        )\
                )\
)
```

Another standard for spectral libraries and transition lists is the `TraML` format. Data from a `.tsv` file can be convereted to `.TraML` using the `TargetedFileConverter` program in Openms.

```
define Traml_Rule
${1} : ${2}
    if not exist $(dir ${1}) mkdir $(dir ${1})
    ${TargetedFileConverter_path} -in ${2} -out ${1} \
    -algorithm:force_invalid_mods
endef

$(foreach TraML,${allTraML},\
        $(eval \
                $(call Traml_Rule,\
                        ${TraML},\
                        $(basename ${TraML}).tsv \
                    )\
            )\
)
```

## Additional Input Files

Some of the programs require attional text files. The rules outlined below define variables which are then writen out to the required files. The text of various small paramater and data files required by the tools outlined above are spesified within the makefile ensuring consistancy. For data re-use and re-processing the sections most likley to be require modification are `SWATH_windows.txt` and the `Xtandem_params.xml`.

### iRT fasta file

```
define outFile_iRTfasta
>Biognosys|iRT-Kit_WR_fusion
LGGNEQVTRYILAGVENSKGTFIIDPGGVIR
GTFIIDPAAVIRGAGSSEPVTGLDAKTPVISGGPYEYR
VEATFGVDESNAKTPVITGAPYEYRDGLDAASYYAPVR
ADVTPADFSEWSKLFLQFGAQGSPFLK
endef

.\ini\irtfusion.fasta :
    $(file > .\ini\irtfusion.fasta,${outFile_iRTfasta})
```

### X!tandem Paramater Files

Search engine X!tandem requires a `taxonomy.xml` file to define the location of the fasta file it will search against. This file must be edited to contain the corect name of the combined forward, reveres and iRT file produced by the make file.

```
define xtandemTaxonomy
<?xml version="1.0"?>
<bioml label="x! taxon-to-file matching list">
    <taxon label="fastaForSearch">
        <file format="peptide" URL="ini/uniprot-proteome3AUP000000589_rev.fasta" />
    </taxon>
</bioml>
endef

.\ini\taxonomy.xml :
    $(file > .\ini\taxonomy.xml,${xtandemTaxonomy})
```

```
define Xtandem_params
<?xml version="1.0"?>
<bioml>
    <note>
    Each one of the parameters for x! tandem is entered as a labeled note node.
    Any of the entries in the default_input.xml file can be over-ridden by
    adding a corresponding entry to this file.
    </note>

    <note type="input" label="list path, default parameters">.\ini\default_input.xml</note>
    <note type="input" label="list path, taxonomy information">.\ini\taxonomy.xml</note>
    <note type="input" label="protein, taxon">fastaForSearch</note>
    <note type="input" label="spectrum, path">spectrumFile</note>
    <note type="input" label="output, path">outFile</note>
    <note type="input" label="output, path hashing">no</note>
    <note>The following 'potential modification motif' entries cover
    the isotopicaly labelled pepCalMix peptides.</note>
    <note type="input" label="residue, modification mass">+57.034@C</note>
    <note type="input" label="residue, potential modification mass">16@M</note>
    <note type="input" label="scoring, maximum missed cleavage sites">1</note>
    <note type="input" label="spectrum, fragment monoisotopic mass error">25</note>
    <note type="input" label="spectrum, parent monoisotopic mass error plus">30</note>
    <note type="input" label="spectrum, parent monoisotopic mass error minus">30</note>
    <note type="input" label="spectrum, parent monoisotopic mass isotope error">yes</note>
    <note type="input" label="spectrum, fragment monoisotopic mass error units">ppm</note>
    <note>The value for this parameter may be 'Daltons' or 'ppm':
    all other values are ignored</note>
    <note type="input" label="spectrum, parent monoisotopic mass error units">ppm</note>
    <note>The value for this parameter may be 'Daltons' or 'ppm':
    all other values are ignored</note>
    <note type="input" label="spectrum, fragment mass type">monoisotopic</note>
    <note type="input" label="scoring, include reverse">no</note>
    <note type="input" label="refine, maximum valid expectation value">0.1</note>
    <note type="input" label="output, maximum valid expectation value">0.1</note>
    <note type="input" label="scoring, x ions">no</note>
    <note type="input" label="scoring, y ions">yes</note>
    <note type="input" label="scoring, z ions">no</note>
```

```
    <note type="input" label="scoring, a ions">no</note>
    <note type="input" label="scoring, b ions">yes</note>
    <note type="input" label="scoring, c ions">no</note>
    <note type="input" label="refine, use potential modifications for full refinement">no</note>
</bioml>
endef

.\ini\Xtandem_params.xml :
    $(file > .\ini\Xtandem_params.xml,${Xtandem_params})
```

```
define xtandemDefautParams
<?xml version="1.0"?>
<?xml-stylesheet type="text\xsl" href="tandem-input-style.xsl"?>
<bioml>
<note>list path parameters</note>
    <note type="input" label="list path, default parameters">default_input.xml</note>
        <note>This value is ignored when it is present in the default parameter
        list path.</note>
    <note type="input" label="list path, taxonomy information">taxonomy.xml</note>
<note>spectrum parameters</note>
    <note type="input" label="spectrum, fragment monoisotopic mass error">0.4</note>
    <note type="input" label="spectrum, parent monoisotopic mass error plus">100</note>
    <note type="input" label="spectrum, parent monoisotopic mass error minus">100</note>
    <note type="input" label="spectrum, parent monoisotopic mass isotope error">yes</note>
    <note type="input" label="spectrum, fragment monoisotopic mass error units">Daltons</note>
    <note>The value for this parameter may be 'Daltons' or 'ppm':
    all other values are ignored</note>
    <note type="input" label="spectrum, parent monoisotopic mass error units">ppm</note>
        <note>The value for this parameter may be 'Daltons' or 'ppm':
        all other values are ignored</note>
    <note type="input" label="spectrum, fragment mass type">monoisotopic</note>
        <note>values are monoisotopic|average </note>

<note>spectrum conditioning parameters</note>
    <note type="input" label="spectrum, dynamic range">100.0</note>
        <note>The peaks read in are normalized so that the most intense peak
        is set to the dynamic range value. All peaks with values of less that
        1, using this normalization, are not used. This normalization has the
        overall effect of setting a threshold value for peak intensities.</note>
    <note type="input" label="spectrum, total peaks">50</note>
        <note>If this value is 0, it is ignored. If it is greater than zero (lets say 50),
        then the number of peaks in the spectrum with be limited to the 50 most intense
        peaks in the spectrum. X! tandem does not do any peak finding: it only
        limits the peaks used by this parameter, and the dynamic range parameter.</note>
    <note type="input" label="spectrum, maximum parent charge">4</note>
    <note type="input" label="spectrum, use noise suppression">yes</note>
    <note type="input" label="spectrum, minimum parent m+h">500.0</note>
    <note type="input" label="spectrum, minimum fragment mz">150.0</note>
    <note type="input" label="spectrum, minimum peaks">15</note>
    <note type="input" label="spectrum, threads">1</note>
    <note type="input" label="spectrum, sequence batch size">1000</note>

<note>residue modification parameters</note>
    <note type="input" label="residue, modification mass">57.022@C</note>
```

```
        <note>The format of this parameter is m@X, where m is the modfication
        mass in Daltons and X is the appropriate residue to modify. Lists of
        modifications are separated by commas. For example, to modify M and C
        with the addition of 16.0 Daltons, the parameter line would be
        +16.0@M,+16.0@C
        Positive and negative values are allowed.
        </note>
    <note type="input" label="residue, potential modification mass"></note>
        <note>The format of this parameter is the same as the format
        for residue, modification mass (see above).</note>
        <note>The format of this parameter is similar to residue, modification mass,
        with the addition of a modified PROSITE notation sequence motif specification.
        For example, a value of 80@[ST!]PX[KR] indicates a modification
        of either S or T when followed by P, and residue and the a K or an R.
        A value of 204@N!{P}[ST]{P} indicates a modification of N by 204, if it
        is NOT followed by a P, then either an S or a T, NOT followed by a P.
        Positive and negative values are allowed.
        </note>

<note>protein parameters</note>
    <note type="input" label="protein, taxon">other mammals</note>
        <note>This value is interpreted using the information in taxonomy.xml.</note>
    <note type="input" label="protein, cleavage site">[RK]|{P}</note>
        <note>this setting corresponds to the enzyme trypsin. The first characters
        in brackets represent residues N-terminal to the bond - the '|' pipe -
        and the second set of characters represent residues C-terminal to the
        bond. The characters must be in square brackets (denoting that only
        these residues are allowed for a cleavage) or french brackets (denoting
        that these residues cannot be in that position). Use UPPERCASE characters.
        To denote cleavage at any residue, use [X]|[X] and reset the
        scoring, maximum missed cleavage site parameter (see below) to something like 50.
        </note>
    <note type="input" label="protein, modified residue mass file"></note>
    <note type="input" label="protein, cleavage C-terminal mass change">+17.002735</note>
    <note type="input" label="protein, cleavage N-terminal mass change">+1.007825</note>
    <note type="input" label="protein, N-terminal residue modification mass">0.0</note>
    <note type="input" label="protein, C-terminal residue modification mass">0.0</note>
    <note type="input" label="protein, homolog management">no</note>
        <note>if yes, an upper limit is set on the number of
        homologues kept for a particular spectrum</note>

<note>model refinement parameters</note>
    <note type="input" label="refine">yes</note>
    <note type="input" label="refine, modification mass"></note>
    <note type="input" label="refine, sequence path"></note>
    <note type="input" label="refine, tic percent">20</note>
    <note type="input" label="refine, spectrum synthesis">yes</note>
    <note type="input" label="refine, maximum valid expectation value">0.1</note>
    <note type="input" label="refine, potential N-terminus modifications">+42.010565@[</note>
    <note type="input" label="refine, potential C-terminus modifications"></note>
    <note type="input" label="refine, unanticipated cleavage">yes</note>
    <note type="input" label="refine, potential modification mass"></note>
    <note type="input" label="refine, point mutations">no</note>
```

```
    <note type="input" label="refine, use potential modifications for full refinement">no</note>
    <note type="input" label="refine, point mutations">no</note>
    <note type="input" label="refine, potential modification motif"></note>
    <note>The format of this parameter is similar to residue, modification mass,
        with the addition of a modified PROSITE notation sequence motif specification.
        For example, a value of 80@[ST!]PX[KR] indicates a modification
        of either S or T when followed by P, and residue and the a K or an R.
        A value of 204@N!{P}[ST]{P} indicates a modification of N by 204, if it
        is NOT followed by a P, then either an S or a T, NOT followed by a P.
        Positive and negative values are allowed.
        </note>

<note>scoring parameters</note>
    <note type="input" label="scoring, minimum ion count">4</note>
    <note type="input" label="scoring, maximum missed cleavage sites">1</note>
    <note type="input" label="scoring, x ions">no</note>
    <note type="input" label="scoring, y ions">yes</note>
    <note type="input" label="scoring, z ions">no</note>
    <note type="input" label="scoring, a ions">no</note>
    <note type="input" label="scoring, b ions">yes</note>
    <note type="input" label="scoring, c ions">no</note>
    <note type="input" label="scoring, cyclic permutation">no</note>
        <note>if yes, cyclic peptide sequence permutation
        is used to pad the scoring histograms</note>
    <note type="input" label="scoring, include reverse">no</note>
        <note>if yes, then reversed sequences are searched at the
        same time as forward sequences</note>
    <note type="input" label="scoring, cyclic permutation">no</note>
    <note type="input" label="scoring, include reverse">no</note>

<note>output parameters</note>
    <note type="input" label="output, log path"></note>
    <note type="input" label="output, message">testing 1 2 3</note>
    <note type="input" label="output, one sequence copy">no</note>
    <note type="input" label="output, sequence path"></note>
    <note type="input" label="output, path">output.xml</note>
    <note type="input" label="output, sort results by">protein</note>
        <note>values = protein|spectrum (spectrum is the default)</note>
    <note type="input" label="output, path hashing">yes</note>
        <note>values = yes|no</note>
    <note type="input" label="output, xsl path">tandem-style.xsl</note>
    <note type="input" label="output, parameters">yes</note>
        <note>values = yes|no</note>
    <note type="input" label="output, performance">yes</note>
        <note>values = yes|no</note>
    <note type="input" label="output, spectra">yes</note>
        <note>values = yes|no</note>
    <note type="input" label="output, histograms">yes</note>
        <note>values = yes|no</note>
    <note type="input" label="output, proteins">yes</note>
        <note>values = yes|no</note>
    <note type="input" label="output, sequences">yes</note>
        <note>values = yes|no</note>
```

```
    <note type="input" label="output, mzid">no</note>
    <note type="input" label="output, one sequence copy">no</note>
        <note>values = yes|no, set to yes to produce only one
        copy of each protein sequence in the output xml</note>
    <note type="input" label="output, results">valid</note>
        <note>values = all|valid|stochastic</note>
    <note type="input" label="output, maximum valid expectation value">0.1</note>
        <note>value is used in the valid|stochastic setting of output, results</note>
    <note type="input" label="output, histogram column width">30</note>
        <note>values any integer greater than 0. Setting this to '1'
        makes cutting and pasting histograms into spread sheet programs easier.</note>
    <note type="description">ADDITIONAL EXPLANATIONS</note>
    <note type="description">Each one of the parameters for X! tandem is entered as a
    labeled note node. In the current version of X!, keep those note nodes
    on a single line.
    </note>
    <note type="description">The presence of the type 'input' is necessary if a
    note is to be considered an input parameter.
    </note>
    <note type="description">Any of the parameters that are paths to files may require
    alteration for a particular installation. Full path names usually cause the least trouble,
            but there is no reason not to use relative path names, if that is the
            most convenient.
    </note>
    <note type="description">Any parameter values set in the 'list path, default parameters'
    file are reset by entries in the normal input file, if they are present. Otherwise,
            the default set is used.
    </note>
    <note type="description">The 'list path, taxonomy information' file must exist.
        </note>
    <note type="description">The directory containing the 'output, path' file must exist:
    it will not be created. </note>
    <note type="description">The 'output, xsl path' is optional: it is only of use if a
    good XSLT style sheet exists. </note>
</bioml>
endef

.\ini\default_input.xml :
    $(file > .\ini\default_input.xml,${xtandemDefautParams})
```

### Retetion Time Index File

The following test in `iRTPeptides.txt` contains the iRT peptide iRT scores.

```
define iRT_File
LGGNEQVTR   -24.92
GAGSSEPVTGLDAK  0.00
VEATFGVDESNAK   12.39
YILAGVENSK  19.79
TPVISGGPYEYR    28.71
TPVITGAPYEYR    33.38
DGLDAASYYAPVR   42.26
ADVTPADFSEWSK   54.62
```

```
GTFIIDPGGVIR    70.52
GTFIIDPAAVIR    87.23
LFLQFGAQGSPFLK  100.00
endef


.\ini\iRTPeptides.txt :
    $(file > .\ini\iRTPeptides.txt,${iRT_File})
```

### SWATH Window Schema File

```
define SWATH_windows
399.5   406
406 412
412 418
418 424
424 430
430 436
436 442
442 448
448 454
454 459
459 464
464 469
469 474
474 479
479 484
484 489
489 494
494 499
499 504
504 509
509 514
514 519
519 524
524 529
529 534
534 539
539 544
544 549
549 554
554 559
559 564
564 569
569 574
574 579
579 584
584 589
589 594
594 599
599 604
604 609
```

```
609 614
614 619
619 624
624 629
629 634
634 639
639 644
644 649
649 654
654 660
660 666
666 672
672 678
678 684
684 690
690 696
696 702
702 708
708 714
714 720
720 726
726 732
732 738
738 744
744 750
750 756
756 763
763 770
770 777
777 784
784 791
791 798
798 805
805 812
812 819
819 826
826 834
834 842
842 850
850 858
858 867
867 876
876 885
885 894
894 903
903 914
914 925
925 936
936 950
950 964
964 978
978 992
992 1011
```

```
1011    1030
1030    1054
1054    1078
1078    1117
1117    1156
1156    1200
1200    1249.5
endef

.\ini\SWATH_windows.txt :
    $(file > .\ini\SWATH_windows.txt,${SWATH_windows})
```

# Conclusion

The above fully documents the makefile used to process the raw mass spectrometry data into spectral libraries for this study. As such we hope that, in the spirit of reproducable research, it will enable direct reproduction of the libraries presented here, and also facilitate re-production with project spesific variations so that data can be re-used in different contexts.

Please contact paper authors for support using any of this code if required.

# References

1. Lam, H. *et al.* Building consensus spectral libraries for peptide identification in proteomics. *Nat Methods* **5,** 873–875 (2008).
